# JPT2 Affects Trophoblast Functions and Macrophage Polarization and Metabolism, and Acts as a Potential Therapeutic Target for Recurrent Spontaneous Abortion

**DOI:** 10.1002/advs.202306359

**Published:** 2024-02-28

**Authors:** Xin Chen, Qian Lin Song, Rui Ji, Jia Yu Wang, Ming Liang Cao, Duan Ying Guo, Yan Zhang, Jing Yang

**Affiliations:** ^1^ Reproductive Medical Center Renmin Hospital of Wuhan University and Hubei Clinic Research Center for Assisted Reproductive Technology and Embryonic Development Wuhan Hubei 430060 China; ^2^ Department of Urology Renmin Hospital of Wuhan University Wuhan Hubei 430060 China; ^3^ Department of Obstetrics and Gynecology Renmin Hospital of Wuhan University Wuhan Hubei 430060 China; ^4^ Department of Gynecology Longgang District People's Hospital of Shenzhen Shenzhen 518172 China

**Keywords:** jupiter microtubule‐associated homolog 2, macrophage metabolism, macrophage polarization, recurrent spontaneous abortion, trophoblast

## Abstract

Recurrent spontaneous abortion (RSA) is a pregnancy‐related condition with complex etiology. Trophoblast dysfunction and abnormal macrophage polarization and metabolism are associated with RSA; however, the underlying mechanisms remain unknown. Jupiter microtubule‐associated homolog 2 (JPT2) is essential for calcium mobilization; however, its role in RSA remains unclear. In this study, it is found that the expression levels of JPT2, a nicotinic acid adenine dinucleotide phosphate‐binding protein, are decreased in the villous tissues of patients with RSA and placental tissues of miscarried mice. Mechanistically, it is unexpectedly found that abnormal JPT2 expression regulates trophoblast function and thus involvement in RSA via c‐Jun *N*‐terminal kinase (JNK) signaling, but not via calcium mobilization. Specifically, on the one hand, JPT2 deficiency inhibits trophoblast adhesion, migration, and invasion by inhibiting the JNK/atypical chemokine receptor 3 axis. On the other hand, trophoblast JPT2 deficiency contributes to M1 macrophage polarization by promoting the accumulation of citrate and reactive oxygen species via inhibition of the JNK/interleukin‐6 axis. Self‐complementary adeno‐associated virus 9‐JPT2 treatment alleviates embryonic resorption in abortion‐prone mice. In summary, this study reveals that JPT2 mediates the remodeling of the immune microenvironment at the maternal–fetal interface, suggesting its potential as a therapeutic target for RSA.

## Graphical abstract

Dysregulated Jupiter microtubule‐associated homolog 2 (JPT2) at the maternal–fetal interface leads to recurrent spontaneous abortion (RSA) development. The present study shows that JPT2 deficiency inhibits trophoblast function. Furthermore, the lack of JPT2 in trophoblasts contributes to M1 macrophage polarization by promoting the accumulation of citrate and reactive oxygen species, which subsequently affect trophoblast function.

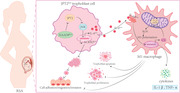

## 1 Introduction

Recurrent spontaneous abortion (RSA), defined as two or more consecutive spontaneous abortions with the same partner, is observed in 5% of all pregnancies and poses a substantial obstacle to normal pregnancy.^[^ ^1^ ^]^ Known etiologies of RSA include infection,^[^ ^2^ ^]^ chromosomal abnormalities,^[^ ^3^ ^]^ endocrine and metabolic dysfunction,^[^ ^4^ ^]^ antiphospholipid syndrome,^[^ ^5^ ^]^ and uterine anatomical abnormalities.^[^ ^6^ ^]^ However, ≈50% of patients have RSA with an unknown etiology, known as unexplained RSA.^[^ ^7^ ^]^ Therefore, the specific causes, regulatory molecules, and underlying mechanisms must be explored further for the prevention and treatment of RSA.

During pregnancy, the placenta acts as a bridge between the mother and fetus, affecting various important functions, such as metabolism, nutrition, and resistance.^[^ ^8^ ^]^ Human extravillous trophoblasts are the cornerstone of the maternal–fetal interface.^[^ ^9^ ^]^ Normal physiological functions are crucial for successful pregnancy.^[^ ^10^ ^]^ Moreover, conversion of the maternal immune system to an immune‐tolerant state is necessary to prevent any attacks on the fetus.^[^ ^11^ ^]^ Therefore, immune cells located at the interface between the placenta and uterus play important roles during pregnancy.^[^ ^12^ ^]^ To maintain the immune tolerance, a subtle crosstalk is established among trophoblasts, immune cells, and decidual stromal cells.^[^ ^13^ ^]^ Dysfunction of fetal‐derived trophoblasts, immune cells, and decidual stromal cells and any abnormal crosstalk among them are associated with various pregnancy complications, including RSA. However, the specific underlying mechanisms remain unknown.

Jupiter microtubule‐associated homolog 2 (JPT2), also known as the hematological and neurologically expressed 1‐like protein, is a newly discovered cancer oncogene that promotes the development of various cancer cells.^[^ ^14^ ^]^ Placental trophoblasts exhibit many cancer‐like characteristics; however, whether JPT2 is involved in the etiopathogenesis of RSA remains ambiguous. Therefore, we hypothesized that JPT2 regulates trophoblast functions and immune tolerance during pregnancy, possibly playing a role in RSA.

In this study, we demonstrated that JPT2 expression levels were downregulated in the villous specimens of patients with RSA and placentas of miscarried mice. RNA‐sequencing (RNA‐seq) analysis revealed that JPT2 deletion inhibited trophoblast adhesion, migration, and invasion by downregulating the c‐Jun *N*‐terminal kinase (JNK)/atypical chemokine receptor 3 (ACKR3) axis. Interestingly, JPT2 deletion in trophoblasts induced M1 polarization and accumulation of reactive oxygen species (ROS) in macrophages by downregulating the JNK/interleukin (IL)‐6 axis. Untargeted metabolomics revealed that citrate accumulation increased the ROS levels in macrophages, further leading to M1 polarization. JPT2 deletion in trophoblasts induces the formation of M1 macrophages, which subsequently regulate the functions of trophoblasts. Overall, our results revealed a novel molecular mechanism of JPT2 dysregulation at the maternal–fetal interface leading to RSA, suggesting its role as a potential therapeutic target for RSA.

## 2 Results

### 2.1 JPT2 Expression Is Decreased in RSA

**Figure 1:**
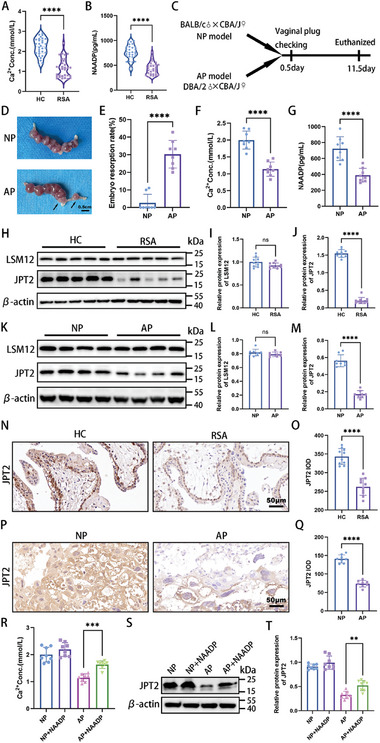
Jupiter microtubule‐associated homolog 2 (JPT2) expression is decreased in recurrent spontaneous abortion (RSA). A,B) Calcium and nicotinic acid adenine dinucleotide phosphate (NAADP) concentrations in the villous tissues of the normal pregnancy (healthy control [HC]) and RSA groups were measured via calcium assay and enzyme‐linked immunosorbent assay (ELISA), respectively (each group: *n* = 30). C) Schematic diagram of the animal experimental protocol. CBA/J females were mated with BALB/c males to establish NP or with DBA/2 males to establish an abortion‐prone pregnancy (AP). Mice were euthanized on day 11.5 of gestation, and embryo resorption rates were calculated. D) Black arrow indicates the embryo resorption. Scale bar, 0.5 cm. E) Determination of embryo resorption rates in the NP and AP groups on gestation day 11.5 (each group: *n* = 8). F,G) Calcium and NAADP concentrations in the placental tissues of mice in the NP and AP groups were measured via calcium assay and ELISA, respectively (each group: *n* = 8). H–M) Protein levels and statistical results of LSM12 and JPT2 in the villous tissues of HC (*n* = 10) and patients with RSA (*n* = 10) and placental tissues of NP mice (*n* = 8) and AP mice (*n* = 8) were determined via western blotting. N–Q) Representative immunohistochemistry (IHC) images and statistical results of JPT2 expression in the villous tissues of HC (*n* = 10) and patients with RSA patients (*n* = 10) and placental tissues of NP mice (*n* = 8) and AP mice (*n* = 8). Scale bar, 50 µm. R–T) Pregnant mice were randomly divided into four groups (NP, NP+NAADP (0.181 mg kg^−1^), AP, and AP+NAADP (0.181 mg kg^−1^) groups) and subsequently assayed for placental calcium concentration and protein levels of JPT2 (each group: *n* = 8). R) Calcium assay kit was used to measure the placental calcium concentration. Protein levels of JPT2 in the placenta of pregnant mice were determined via western blotting, and the statistical results are shown in (S) and (T) (each group: *n* = 8). Error bars indicate the standard deviation (SD) of the mean. Student's *t*‐test was used to assess the differences between two groups, and one‐way analysis of variance (ANOVA) was used to compare the differences among multiple groups. **P < 0.01, ***P < 0.001, and ****P < 0.0001; ns, not significant.

JPT2 was recently identified as a binding protein for nicotinic acid adenine dinucleotide phosphate (NAADP), an important molecule involved in NAADP‐mediated calcium mobilization.^[^ ^15^ ^]^ Calcium homeostasis in the placenta ensures the exchange of nutrients between the mother and fetus, and dysregulation of calcium homeostasis can cause pregnancy‐related diseases.^[^ ^16^ ^]^ Therefore, in this study, we first examined the concentrations of calcium and NAADP in the villous tissues of patients with RSA and healthy controls (HC). As shown in **Figures** **1**A,B and S1A,B (Supporting Information), the concentrations of calcium and NAADP in the villous tissues of patients with RSA were significantly low. Next, we constructed mouse models of normal pregnancy (NP) and abortion‐prone (AP) (Figure 1C). Figure 1D,E shows a significant increase in the embryo resorption rate in the AP group. Subsequently, the intraplacental calcium and NAADP concentrations were monitored again. The results showed that the AP group's placental calcium concentrations (Figure 1F and Figure S1C,D, Supporting Information) and NAADP (Figure 1G) significantly decreased. To verify whether embryo loss in mice is associated with the inhibition of NAADP signaling, we used an NAADP inhibitor (Ned19) to intervene in NP and AP mice. Ned19 increased the embryo resorption rate in both NP and AP mice, but did not increase the embryo resorption rate in NP mice to the extent observed in AP mice without Ned19 (Figure S2A,B, Supporting Information). This suggests that embryo loss is associated with the inhibition of NAADP signaling; however, other mechanisms may also be involved in RSA development.

JPT2 and LSM12 are NAADP‐binding proteins.^[^ ^15c^ ^]^ Therefore, JPT2 and LSM12 expression levels were monitored in this study. As shown in Figure 1H–M, the protein expression levels of JPT2 was significantly decreased in the placental villi of patients with RSA and AP mice, whereas those of LSM12 were not considerably affected. Immunohistochemical results also showed that JPT2 expression levels were significantly reduced in the placentas of patients with RSA (Figure 1N,O) and AP mice (Figure 1P,Q). At the cellular level, JPT2 was expressed in both the nucleus and cytoplasm of trophoblasts. In addition, Pearson's correlation analysis indicated that the expression of JPT2 in the mouse placenta was negatively correlated with the embryo resorption rate (Figure S3A,B, Supporting Information). To further determine whether the downregulation of JPT2 occurs via NAADP‐mediated inhibition of calcium mobilization, we treated mice with NAADP‐AM (membrane‐permeable form of NAADP). NAADP restored the calcium content (Figure 1R and Figure S4A,B, Supporting Information) and expression of JPT2 (Figure 1S,T) in the placenta of AP mice supplemented with NAADP. Finally, we tested the effects of ATP and acetylcholine on the calcium responses in JPT2‐knocked down trophoblasts. JPT2 knockdown inhibited the response of ATP to calcium to some extent but did not alter the response of acetylcholine to calcium (Figure S5E–H, Supporting Information). Therefore, JPT2 acts as a binding protein for NAADP‐mediated calcium mobilization and its downregulation may be a potential mechanism for RSA development.

### 2.2 JPT2 Regulates the Trophoblast Adhesion, Migration, and Invasion

**Figure 2:**
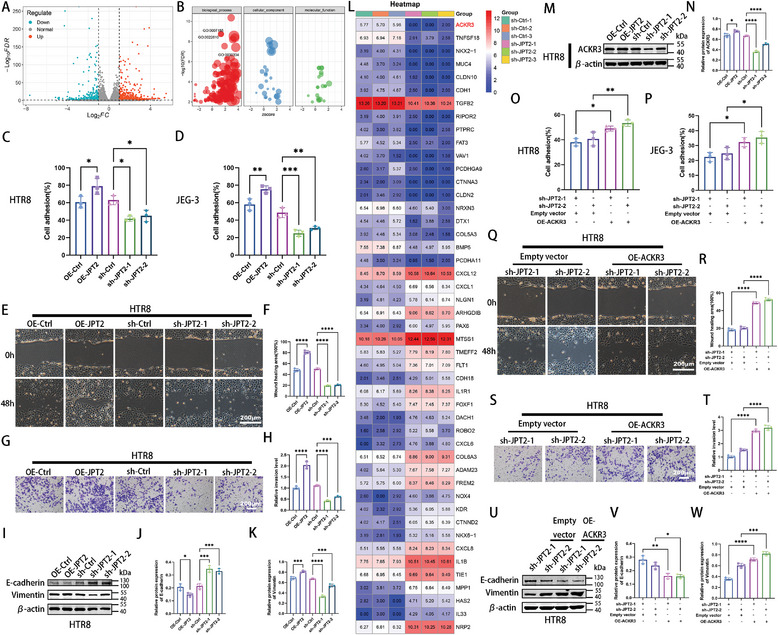
JPT2 regulates the trophoblast adhesion, migration, and invasion. A) Volcano plot shows the differentially expressed genes (DEGs) between the sh‐Ctrl and sh‐JPT2 group HTR8 cells. B) Gene ontology (GO) bubbles showing the functional annotation of differential gene enrichment in (A). GO:0007155: cell adhesion; GO:0022610: biological adhesion; GO:0030334: regulation of cell migration. C,D) Cell adhesion of JPT2‐knockdown or ‐overexpressed HTR8 and JEG‐3 cells. E,F) Migration and quantitative values of HTR8 cells were examined via wound healing assays. Scale bar, 200 µm. G,H) Invasive ability and quantitative values of HTR8 cells were determined via transwell assays. Scale bar, 200 µm. I–K) Protein bands and quantitative values of E‐cadherin and vimentin protein levels in JPT2‐knockdown or ‐overexpressed HTR8 cells. I) Representative protein blot images. J) Quantitative values of E‐cadherin protein levels. K) Quantitative values of vimentin protein levels. L) Heatmap showing the differential expression of genes enriched in “cell adhesion,” “biological adhesion,” and “regulation of cell migration” in (B). M,N) Protein images and quantitative values of ACKR3 protein levels in JPT2‐knockdown or ‐overexpressed HTR8 cells. O–W) Effects of ACKR3 overexpression on trophoblast adhesion, migration, and invasion. O,P) Cell adhesion of HTR8 and JEG‐3 cells. Q,R) Migration ability and quantitative values of HTR8 cells. Scale bar, 200 µm. S,T) Invasive ability and quantitative values of HTR8 cells. Scale bar, 200 µm. U) Representative protein blot images of E‐cadherin and vimentin protein levels in HTR8 cells. V) Quantitative values of E‐cadherin protein levels. W) Quantitative values of vimentin protein levels. Data are represented as the mean ± SD of at least three independent experiments, with each data point representing an independent experiment. Error bars indicate the SD of the mean. One‐way ANOVA was used to compare the differences among multiple groups. *P < 0.05, **P < 0.01, ***P < 0.001, and ****P < 0.0001.

Trophoblasts are the most critical cells in early pregnancy, their normal function is essential for the establishment and maintenance of pregnancy, and their dysfunction can lead to RSA.^[^ ^17^ ^]^ We explored the role of JPT2 in RSA. JPT2 was successfully knocked down in trophoblasts, as shown in Figure S5A–D (Supporting Information). Differential gene expression and functional changes in JPT2 deficiency versus control were analyzed using RNA‐seq. As shown in **Figure** **2**A, the volcano plot shows that 394 genes were down‐regulated and 580 genes were up‐regulated in the sh‐JPT2 group compared with the control group when the significant differential genes were screened with ∣log2FC∣ ≥ 1 and FDR ≤ 0.05. The results of Gene Ontology (GO) analysis (Figure 2B) observed that the biological processes of “cell adhesion,” “biological adhesion,” and “regulation of cell migration” were significantly enriched. The migration and invasive capacity of trophoblasts guarantee placental implantation.^[^ ^18^ ^]^ Cell adhesion and migration are inextricably linked.^[^ ^19^ ^]^ Therefore, we monitored the adhesion, migration, and invasion abilities of HTR8 and JEG‐3 cells following JPT2 knockdown or overexpression. First, JPT2 knockdown and overexpression in HTR8 and JEG‐3 cells were confirmed via western blotting (Figures S5A–D and S6A–D, Supporting Information). The results showed that JPT2 overexpression enhanced the cell adhesion (Figure 2C,D), migration (Figure 2E,F and Figure S6E,F, Supporting Information), and invasion (Figure 2G,H and Figure S6G,H, Supporting Information) abilities of HTR8 and JEG‐3 cells, whereas JPT2 knockdown inhibited these abilities. Next, we monitored the expression of E‐cadherin and vimentin in these cells. E‐cadherin is a critical component of cell adhesion junctions, and its downregulation enhances the migration ability of cells.^[^ ^20^ ^]^ Vimentin is also a marker of epithelial–mesenchymal transition, and the upregulation of vimentin enhances cell migration.^[^ ^21^ ^]^ Western blotting results showed that JPT2 overexpression downregulated E‐cadherin protein expression in HTR8 (Figure 2I–K) and JEG‐3 cells (Figure S6I–K, Supporting Information) and upregulated vimentin protein expression.

During NAADP‐mediated calcium mobilization, NAADP signaling activates two‐pore channels (TPCs) on endolysosomes to trigger Ca^2+^ release.^[^ ^15b,c^ ^]^ Therefore, to explore whether JPT2 affects trophoblast adhesion, migration, and invasion through TPCs, we knocked down TPC1 and TPC2 in HTR8 and JEG3 cells (Figure S7A,B, Supporting Information). Although the knockdown of TPC1 and TPC2 decreased the calcium content in HTR8 and JEG‐3 cells (Figure S7C,D, Supporting Information), it did not affect the adhesion (Figure S7E,F, Supporting Information), migration (Figure S7G,H, Supporting Information), and invasion (Figure S7I,J, Supporting Information) of HTR8 and JEG‐3 cells. This suggests that JPT2 may affect changes in intracellular calcium levels in trophoblasts through TPCs. However, alterations in trophoblast behavior by JPT2 may not be related to TPCs.

Therefore, to further explore the mechanism by which JPT2 affects trophoblast adhesion, migration, and invasion, we analyzed genes enriched in “cell adhesion,” “biological adhesion,” and “regulation of cell migration” and found that ACKR3 was the most differentially expressed gene (Figure 2L). Subsequently, western blotting showed that JPT2 overexpression upregulated ACKR3 expression in HTR8 (Figure 2M,N) and JEG‐3 (Figure S8A,B, Supporting Information) cells, whereas knockdown of JPT2 downregulated ACKR3 protein expression. ACKR3 promotes the migration and invasion of various cell types.^[^ ^22^ ^]^ To further verify the role of ACKR3, overexpression of the ACKR3 gene was performed in HTR8 and JEG‐3 cells (Figure S8C–E, Supporting Information). Subsequently, further results showed that ACKR3 overexpression reversed the cell adhesion (Figure 2O,P), migration (Figure 2Q,R and Figure S8F,G, Supporting Information), and invasion (Figure 2S,T and Figure S8H,I, Supporting Information) abilities of HTR8 and JEG‐3 cells inhibited by JPT2 deficiency. Moreover, western blotting results showed that ACKR3 overexpression reversed the protein expression of E‐cadherin upregulated by JPT2 deficiency, and vimentin protein expression was downregulated by JPT2 deficiency in HTR8 (Figure 2U–W) and JEG‐3 (Figure S8J–L, Supporting Information) cells. However, co‐immunoprecipitation revealed that JPT2 and ACKR3 did not directly interact with each other in HTR8 and JEG‐3 cells (Figure S9A,B, Supporting Information), suggesting that JPT2 may influence the expression of ACKR3 through other pathways to further regulate trophoblast function. These data indicate that JPT2 deletion leads to decreased trophoblast adhesion, migration, and invasion, likely mediated by the downregulation of ACKR3.

### 2.3 JPT2‐Deficient Trophoblasts Promote M1 Polarization and ROS Accumulation in Macrophages

**Figure 3:**
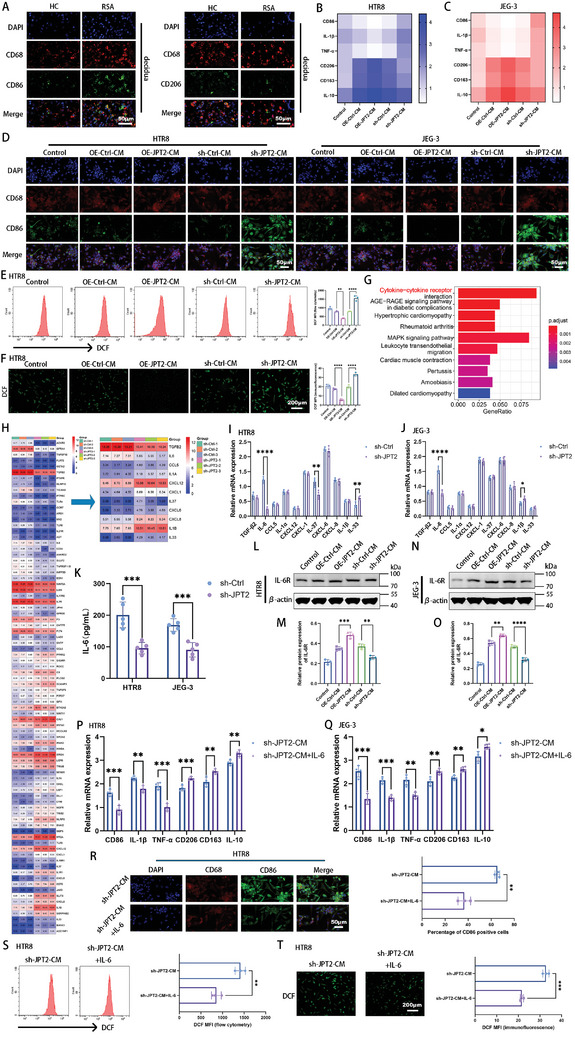
JPT2‐deficient trophoblast‐derived conditional medium promotes M1 polarization and reactive oxygen species (ROS) accumulation in macrophages. A) Co‐localization of CD68 and CD86 or CD68 and CD206 in the decidual tissues of patients in the HC and RSA groups (each group: *n* = 10). Scale bar, 50 µm. B,C) mRNA expression levels of M1 (CD86, IL‐1β, and tumor necrosis factor [TNF]‐*α*) and M2 (CD206, CD163, and IL‐10) macrophage markers were measured in macrophages after intervention with the conditioned medium derived from HTR8 and JEG‐3 cells via qPCR. D) Representative fluorescence images of CD68 and CD86 in macrophages treated with the conditioned medium of HTR8 and JEG‐3 cells. Scale bar, 50 µm. E,F) Flow cytometry and representative fluorescence images showing the fluorescence intensity of dichlorofluorescein (DCF) in macrophages treated with the conditioned medium of HTR8 cells. Scale bar, 200 µm. G) Kyoto encyclopedia of genes and genomes (KEGG) pathway enrichment analysis of HTR8 cells in the sh‐Ctrl and sh‐JPT2 groups. H) Heatmap showing the differential expression of genes enriched in cytokine–cytokine receptor‐associated differential genes. Left panel: cytokine–cytokine receptor‐associated differential genes. Right panel: selected cytokines and chemokines. I,J) Differential expression of cytokines and chemokines selected from (H) in JP2‐knocked down HTR8 and JEG‐3 cells were determined via qPCR. K) ELISA revealed the expression levels of IL‐6 in JPT2‐knocked down HTR8 and JEG‐3 cell supernatants. L–O) Protein expression levels of IL‐6R in macrophages after intervention with the conditioned medium of HTR8 and JEG‐3 cells. Representative protein blot images are shown in (L) and (N). Statistical values of IL‐6R protein levels are shown in (M) and (O). P,Q) Macrophages were treated with the supernatants of HTR8 and JEG‐3 cells with or without IL‐6 (50 ng mL^−1^), and mRNA expression levels of M1 (CD86, IL‐1β, and TNF*‐α*) and M2 (CD206, CD163, and IL‐10) macrophage markers were determined via qPCR. R) Representative fluorescence images of CD68 and CD86 and quantitative values of the percentage of CD86‐positive cells after treatment of macrophages with the supernatants of HTR8 cells with or without IL‐6 (50 ng mL^−1^). Scale bar, 50 µm. S,T) Flow cytometry and representative fluorescence images show the fluorescence intensity of DCF in macrophages treated with the conditioned medium of HTR8 cells with or without IL‐6 (50 ng mL^−1^). Scale bar, 200 µm. Data are represented as the mean ± SD of at least three independent experiments, with each data point representing an independent experiment. Error bars indicate the SD of the mean. Student's *t*‐test was used to assess the differences between two groups, and one‐way ANOVA was used to compare the differences among multiple groups. *P < 0.05, **P < 0.01, ***P < 0.001, and ****P < 0.0001.

Intercommunication between trophoblasts and macrophages is an essential part of RSA.^[^ ^23^ ^]^ M1 macrophages are predominant over M2 macrophages in the RSA.^[^ ^24^ ^]^ In the present study, M1‐like macrophages with CD68^+^CD86^+^ were higher in women with RSA than in HC, whereas M2‐like macrophages with CD68^+^CD206^+^ were lower in women with RSA than in HC (**Figures** **3**A and S10A, Supporting Information). These data suggest that abnormal M1‐like macrophage polarization is exhibited in the decidua of patients with RSA. Therefore, we investigated whether JPT2 deficiency in trophoblasts affects the polarization state of macrophages. First, M0 macrophages were generated by treating THP‐1 cells with 100 ng mL^−1^ phorbol 12‐myristate 13‐acetate for 24 h. Cell morphology (Figure S10B, Supporting Information) and quantitative polymerase chain reaction (qPCR) results (Figure S10C, Supporting Information) showed successful induction of M0 macrophages. qPCR revealed that the conditioned medium of JPT2‐overexpressing HTR8 (Figure 3B) and JEG‐3 (Figure 3C) cells downregulated the expression levels of M1 macrophage markers (CD86, IL‐1β, and tumor necrosis factor [TNF]‐α) and upregulated the expression levels of M2 macrophage markers (CD206, CD163, and IL‐10), while the opposite was true in JPT2 deficiency. The results of immunofluorescence double staining for CD68 and CD86 also confirmed the polarized state of macrophages (Figure 3D and Figure S10D,E, Supporting Information). We also used flow cytometry to determine the polarization status of the macrophages. The results showed that the conditioned medium of JPT2 deficiency trophoblasts promoted M1 polarization in macrophages and that the conditioned medium of JPT2 overexpression trophoblasts inhibited M1 macrophage polarization (Figure S10F,G, Supporting Information). As is well known, the accumulation of ROS in macrophages is closely related to their polarization state.^[^ ^25^ ^]^ As shown in Figure 3E,F and Figure S10H,I (Supporting Information), conditioned medium following JPT2 overexpression in HTR‐8 and JEG3 cells inhibited ROS accumulation in macrophages, whereas conditioned medium following JPT2 knockdown enhanced ROS accumulation in macrophages.

Next, we attempted to explain the mechanism of macrophage response. As shown in Figure 2, JPT2 regulates trophoblast function through ACKR3. Therefore, we further investigated whether the differential expression of JPT2 in trophoblasts affects macrophage polarization status via ACKR3. We knocked down JPT2 and overexpressed ACKR3 genes in HTR8 and JEG‐3 cells and monitored the macrophage polarization status after culturing the macrophages in trophoblast‐conditioned medium. Overexpression of ACKR3 did not affect the macrophage M1 polarization enhanced by JPT2 knockdown (Figure S11A–J, Supporting Information). This suggests the presence of other mechanisms leading to macrophage M1 polarization promoted by JPT2 deficiency in trophoblasts.

Kyoto encyclopedia of genes and genomes (KEGG) analysis and heat maps were used to analyze the potential mechanisms by which trophoblasts affect macrophage polarization. As shown in Figure 3G, cytokine–cytokine receptor signaling was the most significantly enriched function. Trophoblasts can secrete cytokines that alter the phenotype of decidual macrophages.^[^ ^26^ ^]^ Subsequently, the heatmap (Figure 3H) shows differentially expressed genes enriched in cytokine–cytokine receptor signaling, where cytokines are magnified. Next, the qPCR results showed the mRNA expression of these cytokines in HTR8 (Figure 3I and Figure S12A, Supporting Information) and JEG‐3 cells (Figure 3J and Figure S12B, Supporting Information), where IL‐6 was significantly differentially expressed in both HTR8 and JEG‐3 cells. Enzyme‐linked immunosorbent assay (ELISA) further revealed that the expression of IL‐6 in the supernatants of JPT2‐knocked down or ‐overexpressed HTR8 cells and JEG3 cells were decreased and increased, respectively (Figure 3K and Figure S12C,D, Supporting Information). Therefore, IL‐6 is likely critical for JPT2 deficiency in trophoblasts to promote M1 polarization in macrophages. IL‐6 promotes the M2 polarization of macrophages in solid tumors and inflammatory environments.^[^ ^27^ ^]^ IL‐6R is an essential receptor for IL‐6 to perform its function.^[^ ^28^ ^]^ Therefore, IL‐6R was detected in the macrophages. Western blotting results showed that conditioned medium derived from JPT2 overexpressed HTR8 cells and JEG‐3 cells promoted the protein expression of IL‐6R in macrophages, while the opposite effect was observed in JPT2 deficient HTR8 and JEG‐3 cells (Figure 3L,M and Figure 3N,O). To further confirm whether IL‐6 is critical for the trophoblast‐derived conditioned medium to affect macrophage polarization, exogenous IL‐6 was added to the conditioned medium. As shown in Figure 3P and Figure 3Q, IL‐6 supplementation reversed the expression of M1 macrophage markers (CD86, IL‐1β, and TNF‐α) up‐regulated and M2 macrophage markers (CD206, CD163, and IL‐10) down‐regulated by JPT2‐deficient HTR8 cells and JEG‐3 cell‐derived conditioned medium. Double immunofluorescence staining and flow cytometry showed similar results (Figure 3R and Figure S12E–G, Supporting Information). Moreover, the ROS assay results (Figure 3S,T and Figure S12H,I, Supporting Information) showed that IL‐6 supplementation rescued the increased ROS accumulation in macrophages due to JPT2 deficiency in HTR‐8 and JEG3 cells. These data indicate that conditioned medium derived from trophoblasts with JPT2 deficiency can promote M1 polarization and ROS accumulation in macrophages, which may be exerted by inhibiting IL‐6 secretion.

### 2.4 JNK Signaling Is Critical for JPT2‐Mediated Regulation of Trophoblast Functions and Promotion of M1 Polarization and ROS Accumulation in Macrophages

**Figure 4:**
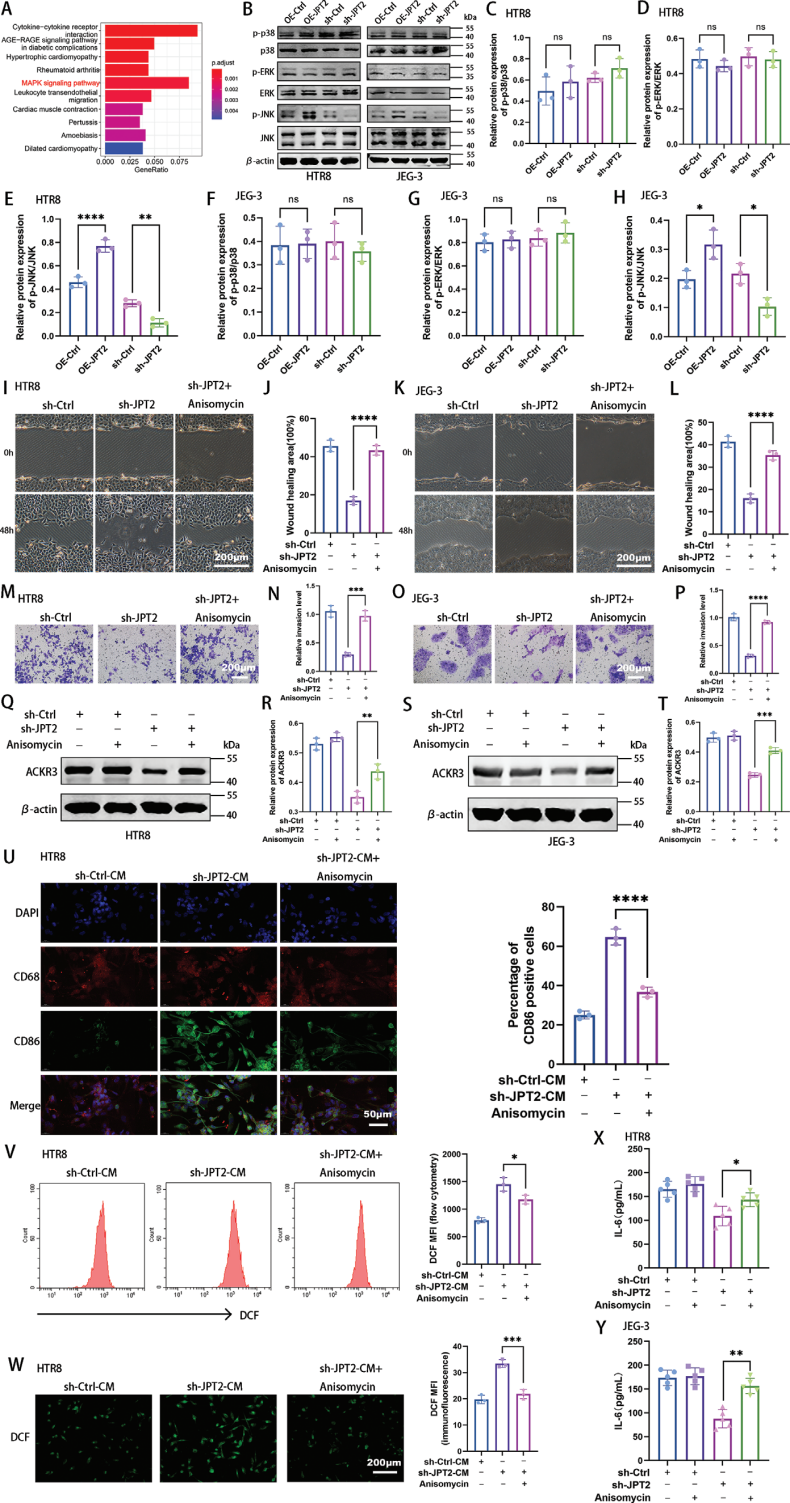
c‐Jun *N*‐terminal kinase (JNK) signaling is critical for JPT2‐mediated regulation of trophoblast functions and promotion of M1 polarization and ROS accumulation in macrophages. A) KEGG pathway enrichment analysis of HTR8 cells in the sh‐Ctrl and sh‐JPT2 groups. B–H) Representative protein bands and statistical values of p‐p38, p38, phospho‐extracellular signal‐regulated kinase (p‐ERK), ERK, p‐JNK, and JNK in JPT2‐knocked down or ‐overexpressed HTR8 and JEG‐3 cells. I–P) Changes in the migration and invasion of trophoblasts after treatment with the JNK activator, anisomycin (10 µm). I–L) Migration ability and quantitative values of HTR8 and JEG‐3 cells. Scale bar, 200 µm. M–P) Invasive ability and quantitative values of HTR8 and JEG‐3 cells. Scale bar, 200 µm. Representative protein bands and statistical values of ACKR3 in HTR8 and JEG‐3 cells after treatment with the JNK activator, anisomycin (10 µm), are shown in (Q–T). After the pretreatment of trophoblasts with JNK activator anisomycin (10 µm), the supernatant of trophoblasts was collected and used to treat macrophages. U) Representative fluorescence images of CD68 and CD86 and quantitative values of the percentage of CD86‐positive cells in macrophages. Scale bar, 50 µm. V,W) Flow cytometry and representative fluorescence images showing the fluorescence intensity of DCF in macrophages treated with the conditioned medium of HTR8 cells. Scale bar, 200 µm. X,Y) ELISA revealed the expression levels of IL‐6 in the supernatants of HTR8 and JEG‐3 cells after the treatment of trophoblasts with the JNK activator, anisomycin (10 µm). Data are represented as the mean ± SD of at least three independent experiments, with each data point representing an independent experiment. Error bars indicate the SD of the mean. One‐way ANOVA was used to compare the differences among multiple groups. *P < 0.05, **P < 0.01, ***P < 0.001, and ****P < 0.0001; ns, not significant.

Next, the effect of JPT2 deficiency on ACKR3 and IL‐6, thus inhibiting trophoblast migration and invasion and promoting macrophage M1 polarization, needs to be explored. As shown in **Figure** **4**A, the mitogen‐activated protein kinase (MAPK) signaling pathway was significantly enriched. MAPK signaling is involved in various biological activities, such as cell proliferation, differentiation, migration, and apoptosis. Mammals contain three major MAPK groups: extracellular signal‐regulated kinase (ERK), JNK, and P38MAPK.^[^ ^29^ ^]^ Therefore, we examined the MAPK signaling pathways. Western blotting results showed that JPT2 overexpression enhanced JNK phosphorylation in HTR8 and JEG‐3 cells and that JPT2 deficiency inhibited JNK phosphorylation in HTR8 and JEG‐3 cells, whereas the phosphorylation of P38 and ERK did not change significantly (Figure 4B–H). Reversal assays were performed using anisomycin, a JNK activator. Cell adhesion, wound healing, and transwell assays showed that anisomycin reversed the cell adhesion (Figure S13A, Supporting Information), migration (Figure 4I–L), and invasion (Figure 4M–P) abilities of HTR8 and JEG‐3 cells inhibited by JPT2 deficiency. Next, we investigated whether JNK signaling affects trophoblast adhesion, migration, and invasion in relation to ACKR3 expression. Western blotting showed that anisomycin reversed ACKR3 protein expression in JPT2‐deficient suppressed HTR8 cells (Figure 4Q,R) and JEG‐3 cells (Figure 4S,T). Macrophage polarization status was also monitored. QPCR results (Figure S13B, Supporting Information), double immunofluorescence (Figure 4U and Figure S13C, Supporting Information), and flow cytometry (Figure S13D,E, Supporting Information) showed that anisomycin reversed the M1 polarization of macrophages promoted by JPT2 deficient trophoblasts. The results of the ROS assay (Figure 4V,W and Figure S13F,G, Supporting Information) showed that anisomycin rescued the increase in ROS content in macrophages caused by JPT2 deficiency in trophoblasts. Next, the relationship between JNK signaling and IL‐6 expression was investigated. ELISA results showed that anisomycin reversed the reduction of IL‐6 concentration in the supernatant of HRT8 (Figure 4X) and JEG‐3 cells (Figure 4Y) with JPT2 deficiency. Taken together, these results suggest that JPT2 deficiency inhibits trophoblast function and promotes M1 polarization, and that the accumulation of ROS in macrophages is likely exerted by suppressing ACKR3 and IL‐6 expression through inhibition of JNK phosphorylation.

### 2.5 JPT2‐Deficient Trophoblasts Promote M1 Polarization and ROS Accumulation by Enhancing the Citrate Production in Macrophages

**Figure 5:**
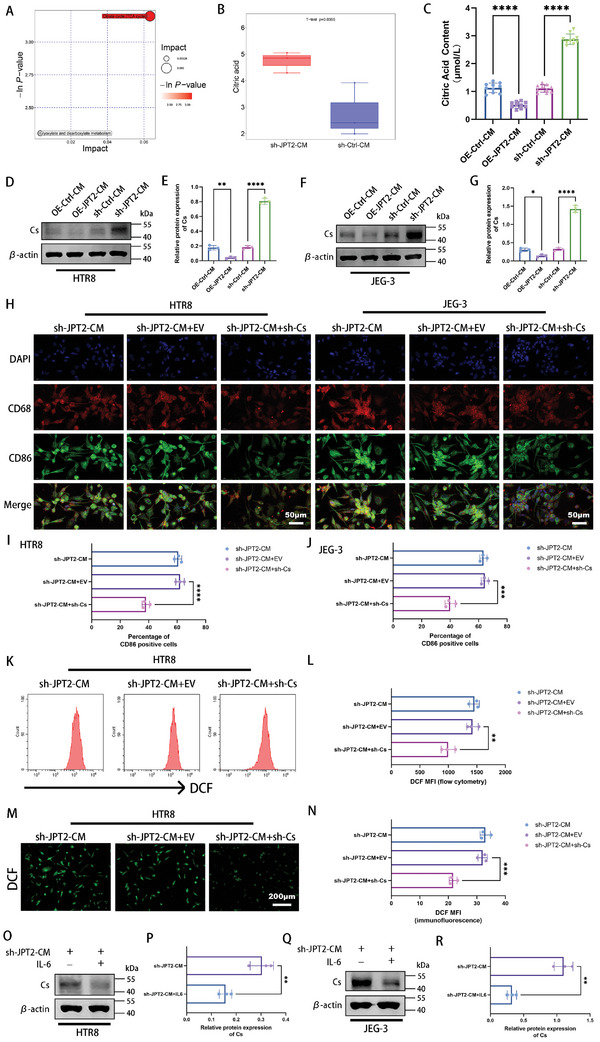
JPT2‐deficient trophoblasts promote M1 polarization and ROS accumulation by enhancing the citrate production in macrophages. A) Metabolic pathway bubble diagram showing the pathways of differential metabolite enrichment. B) Citric acid levels in macrophages. C) Citric acid levels in macrophages after intervention with HTR8 cell‐derived conditioned medium were determined via citric acid content assay. D–G) Protein expression of citrate synthase (Cs) in macrophages after intervention with the HTR8 and JEG‐3 cell‐derived conditioned medium. Representative protein bands of Cs are shown in (D) and (F). Statistical results of protein expression of Cs are shown in (E) and (G). H–J) After treatment of macrophages with empty vector (EV) and sh‐Cs plasmids, the macrophages were intervened with the culture supernatants of HTR8 and JEG‐3 cells. H) Representative fluorescence images of CD68 and CD86 in macrophages. Scale bar, 50 µm. I,J) Quantitative values of the percentage of CD86‐positive cells. K–N) Flow cytometry and representative fluorescence images showing the fluorescence intensity of DCF in macrophages treated with the conditioned medium of HTR8 cells. Scale bar, 200 µm. O–R) Intervention of macrophages with the conditioned medium of HTR8 and JEG‐3 cells with or without IL‐6 (50 ng mL^−1^). Protein expression levels of Cs in macrophages were determined via western blotting. Data are represented as the mean ± SD of at least three independent experiments, with each data point representing an independent experiment. Error bars indicate the SD of the mean. Student's *t*‐test was used to assess the differences between two groups, and one‐way ANOVA was used to compare the differences among multiple groups. *P < 0.05, **P < 0.01, ***P < 0.001, and ****P < 0.0001.

Macrophage metabolic activity is an essential factor regulating their polarization and function. Energy and metabolites required for macrophage polarization and other functions require the balance and activity of intra‐macrophage metabolism.^[^ ^30^ ^]^ Therefore, we investigated whether the effect of JPT2‐deficient trophoblasts on macrophage polarization is related to macrophage metabolism. Untargeted metabolomics was used to analyze the metabolic state of macrophages. After scanning for untargeted metabolomics, 8812 features were identified. The principal component analysis score scatter plot showed a significant difference between the sh‐Ctrl–CM and sh‐JPT2–CM groups (Figure S14A, Supporting Information). Next, we set the threshold criteria as *P* < 0.05, variable importance in projection >1, fold change >1.5 and <0.67 to screen differential metabolites. The metabolite classification results showed that lipids and lipid‐like molecules, organoheterocyclic compounds, and organic acids and their derivatives were the top three metabolite categories, accounting for 39.357%, 11.647%, and 7.631% of all metabolites, respectively (Figure S14B, Supporting Information). Volcano plots showed the differential metabolites, where differential metabolites with levels B(i) and B(ii) were labeled (Figure S14C, Supporting Information). The heatmap further showed the differential expression of the labeled differential metabolites (Figure S14D, Supporting Information). Next, the metabolic pathways enriched by these differential metabolites were analyzed. Bubble plots revealed the citrate (TCA) cycle as the most significant metabolic pathway (**Figure** **5**A). Heat map showed the differential expression and metabolite amounts of all differential metabolic pathways (Figure S14E, Supporting Information). KEGG pathway map further revealed that citrate was the key differential metabolite in the citrate cycle (Figure S14F, Supporting Information). As shown in Figure 5B,C, the citrate content was significantly higher in the sh‐JPT2–CM group than in the sh‐Ctrl–CM group. Citrate synthase (Cs) is a critical enzyme in citric acid synthesis.^[^ ^31^ ^]^ Therefore, Cs in macrophages was monitored. The results showed that JPT2‐deficient HTR8 cells and JEG‐3 cell‐derived conditioned medium promoted the mRNA and protein expression of Cs in macrophages, whereas JPT2 overexpression had the opposite effect (Figure S15A,B, Supporting Information, and Figure 5D–G). Citric acid plays essential roles in many cellular metabolic and immune response processes, and citrate accumulation contributes to the maintenance of M1 polarization in macrophages.^[^ ^32^ ^]^ Inflammatory macrophages strongly express citrate synthase.^[^ ^33^ ^]^ Therefore, Cs knockdown in macrophages (Figure S15C,D, Supporting Information) was used for reversal experiments to confirm the effect of citrate in the role of trophoblasts on macrophage polarization. Dual immunofluorescence results for CD68 and CD86, qPCR results, and flow cytometry showed that the knockdown of Cs in macrophages significantly reversed the enhanced M1 polarization of macrophages by JPT2‐deficient HTR8 and JEG‐3 cells (Figure 5H–J and Figure S15E–H, Supporting Information). Moreover, knockdown of Cs in macrophages reversed the increased ROS content in macrophages due to JPT2 deficiency in trophoblasts (Figure 5K–N and Figure S15I,J, Supporting Information). Based on these results, we found that JPT2‐deficient trophoblast‐derived conditioned medium promoted M1 macrophages by inhibiting IL‐6. Therefore, exogenous IL‐6 was added to the conditioned medium and the expression of Cs was monitored again. Addition of IL‐6 reduced the increase in Cs protein levels in macrophages resulting from macrophages cultured in JPT2‐deficient trophoblast‐conditioned medium (Figure 5O–R). Similar results were obtained using qPCR (Figure S15K,L, Supporting Information). These data suggest that JPT2 deficiency in trophoblasts increases macrophage citrate production by inhibiting IL‐6 secretion, thereby promoting ROS accumulation and leading to macrophage M1 polarization.

### 2.6 Macrophages Educated by JPT2‐Deficient Trophoblasts Inhibit the Trophoblast Functions and Promote Trophoblast Apoptosis

**Figure 6:**
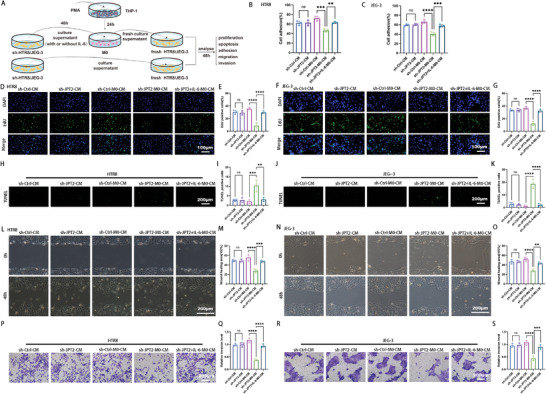
Macrophages educated by JPT2‐deficient trophoblasts inhibit the trophoblast proliferation, adhesion, migration, and invasion and promote trophoblast apoptosis. A) Schematic diagram of the cell treatment process. THP‐1 cells were converted into M0 macrophages by phorbol 12‐myristate 13‐acetate (PMA). The supernatants (with or without IL‐6 [50 ng mL^−1^]) of trophoblasts from the sh‐Ctrl and sh‐JPT2 groups were used to culture M0 macrophages or fresh trophoblasts. The educated macrophages were cultured with the fresh medium, and the supernatant of M0 macrophages was used to culture the fresh trophoblasts. Finally, the proliferation, adhesion, migration, and invasion of these fresh trophoblasts were determined. B,C) Cell adhesion of HTR8 and JEG‐3 cells. D–G) Proliferation of HTR8 and JEG‐3 cells was assessed via the 5‐ethynyl‐2′‐deoxyuridine (EdU) assay. Scale bar, 100 µm. H–K) Apoptosis of HTR8 and JEG‐3 cells was assessed via TdT‐mediated dUTP nick‐end labeling (TUNEL) staining. Scale bar, 200 µm. L–O) Migration ability of HTR8 and JEG‐3 cells was assessed via wound healing assays. Scale bar, 200 µm. P–S) Representative images and quantitative values of invasion of HTR8 and JEG‐3 cells. Scale bar, 200 µm. Data are represented as the mean ± SD of at least three independent experiments, with each data point representing an independent experiment. Error bars indicate the SD of the mean. One‐way ANOVA was used to compare the differences among multiple groups. **P < 0.01, ***P < 0.001, and ****P < 0.0001; ns, not significant.

The signal exchange between trophoblasts and macrophages is usually interactive.^[^ ^34^ ^]^ Therefore, we investigated whether JPT2‐deficient trophoblast‐conditioned medium intervention in macrophages affected trophoblasts. The experimental flow is illustrated in **Figure** **6**A. The proliferative capacity of trophoblasts is essential for maintaining normal pregnancy.^[^ ^35^ ^]^ The results demonstrated that JPT2‐deficient trophoblast‐derived conditioned medium further inhibited trophoblast proliferation (Figure 6H–K), adhesion (Figure 6B,C), migration (Figure 6L–O), and invasive capacity (Figure 6P–S) as well as promoted apoptosis (Figure 6D–G). And supplementation of IL‐6 reversed these inhibitory effects (Figure 6B–S). To exclude the effects of trophoblast autocrine on trophoblasts themselves, we directly treated fresh trophoblasts with the conditioned medium derived from JPT2‐knocked down trophoblasts. It did not have significant effects on trophoblast proliferation, adhesion, migration, invasion, and apoptosis (Figure 6A–S). These data suggest that JPT2 deficiency in trophoblasts affects macrophages via IL‐6 and educated macrophages, thus inhibiting trophoblast proliferation, adhesion, migration, and invasion and promoting trophoblast apoptosis.

### 2.7 ScAAV‐JPT2 Treatment Alleviates Embryo Loss in a Mouse Miscarriage Model

**Figure 7:**
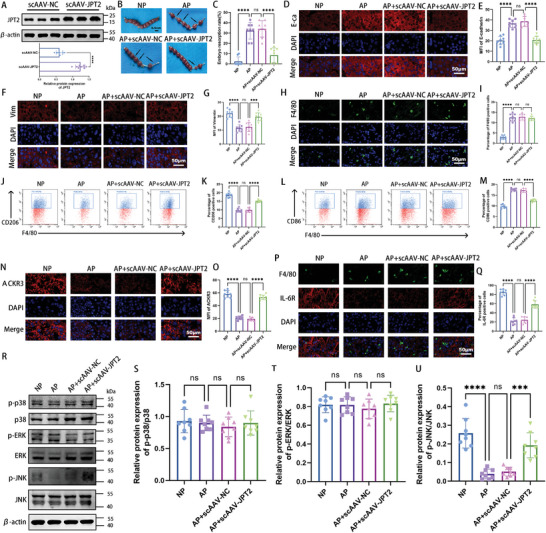
JPT2 treatment alleviates embryo loss in a mouse miscarriage model. A) Representative protein bands and statistical values of JPT2 in the placenta of pregnant mice treated with scAAV‐JPT2 or scAAV‐NC (each group: *n* = 8). B,C) Embryo resorption rates of each group of pregnant mice were measured at day 11.5 of gestation (each group: *n* = 8). B) Black arrows indicate embryo resorption. Scale bar, 0.5 cm. C) Statistical results of embryo resorption rate (each group: *n* = 8). D) Representative fluorescence images of E‐cadherin (E‐cad) at the placental interface of mice. Scale bar, 50 µm. E) Quantitative values of E‐cadherin mean fluorescence intensity (MFI; each group: *n* = 8). F,G) Representative fluorescence images and quantitative values of vimentin (Vim) at the placental interface of mice (each group: *n* = 8). Scale bar, 50 µm. H,I) Representative fluorescence images and quantitative values of the percentage of F4/80‐positive cells at the placental interface of mice (each group: *n* = 8). Scale bar, 50 µm. J,K) Representative flow cytometry results and quantitative values of CD206 in the decidua macrophages of mice (each group: *n* = 8). L,M) Representative flow cytometry results and statistical values of CD86 in the decidua macrophages of mice (each group: *n* = 8). N,O) Immunostaining and statistical results of ACKR3 at the placental interface of mice (each group: *n* = 8). Scale bar, 50 µm. P,Q) Immunostaining statistical results for IL‐6R in the decidua macrophages of mice (each group: *n* = 8). Scale bar, 50 µm. R–U) Representative protein bands and statistical values of p‐p38, p38, p‐ERK, ERK, p‐JNK, and JNK at the placental interface of mice (each group: *n* = 8). Error bars indicate the SD of the mean. Student's *t*‐test was used to assess the differences between two groups, and one‐way ANOVA was used to compare the differences among multiple groups. ***P < 0.001 and ****P < 0.0001; ns, not significant.

To obtain further evidence for the role of JPT2 in RSA, we performed in vivo animal experiments. First, scAAV‐JPT2 was used to enhance JPT2 expression in the mouse placenta. As shown in **Figure** **7**A, the protein expression of JPT2 in the placenta was successfully enhanced by scAAV‐JPT2 injection. As shown in Figure 7B,C, scAAV‐JPT2 significantly reversed the embryo resorption rate. Immunofluorescence results showed that protein expression of E‐cadherin was significantly upregulated, and protein expression of vimentin was significantly downregulated in the placentas of AP group mice compared to that in NP group mice (Figure 7D–G). scAAV‐JPT2 significantly reversed the protein expression of upregulated E‐cadherin (Figure 7D,E) and downregulated vimentin (Figure 7F,G) in the placentas of the AP group mice. Next, the polarization status of macrophages in the placenta was monitored. The immunofluorescence results showed that the percentage of F4/80‐positive cells in the placentas of mice in the AP group was significantly higher than that in the NP group, whereas scAAV‐JPT2 treatment did not alter the percentage of F4/80‐positive cells in the placentas of mice in the AP group (Figure 7H,I). The percentage of CD206‐ or CD86‐positive cells in the placentas of mice was examined via flow cytometry. We found that the levels of the M2 macrophage marker, CD206, were significantly downregulated, whereas those of the M1 macrophage marker, CD86, were significantly upregulated in the mouse placentas of the AP group compared to those in the mouse placentas of the NP group. However, scAAV‐JPT2 treatment significantly reversed the downregulation of CD206 expression and upregulated CD86 expression in the placentas of AP group mice (Figure 7J–M). These results suggest that scAAV‐JPT2 treatment contributes to the conversion of the M1 to M2 phenotype in the placentas of AP group mice. As shown in Figure 7N–Q, ACKR3 expression and the percentage of IL‐6R–positive macrophages were significantly reduced in the placenta of mice in the AP group, whereas scAAV‐JPT2 treatment significantly reversed the reduced ACKR3 expression and the percentage of IL‐6R–positive macrophages in the placenta of mice in the AP group. The expression of MAPK signaling in the mouse placenta was further monitored. As shown in Figure 7R–U, the phosphorylation of JNK was significantly decreased in the placentas of AP mice compared to that in NP mice, and scAAV‐JPT2 treatment reversed the reduced phosphorylation of JNK in the placentas of AP mice. However, the phosphorylation of P38 and ERK did not change significantly. In Figure 1S,T, we found that treatment with NAADP significantly reversed the downregulation of JPT2 in the placenta of AP group mice; therefore, we also monitored whether NAADP injection in AP mice had an effect similar to that of scAAV‐JPT2. The results showed that NAADP effectively reduced the embryo resorption rate (Figure S16A,B, Supporting Information), increased the migration and invasion of trophoblasts (Figure S16C–F, Supporting Information), promoted M2 polarization, and inhibited M1 polarization of placental macrophages (Figure S16G–J, Supporting Information) in the AP group mice; however, the therapeutic effect was insufficient compared with that of scAAV‐JPT2. These results suggest that JPT2 treatment alleviates embryonic loss, attenuates diminished trophoblast invasion, and inhibits macrophage M1 polarization in mouse models of miscarriage.

### 2.8 Activation of JNK Signaling Is Vital for RSA Treatment via JPT2

**Figure 8:**
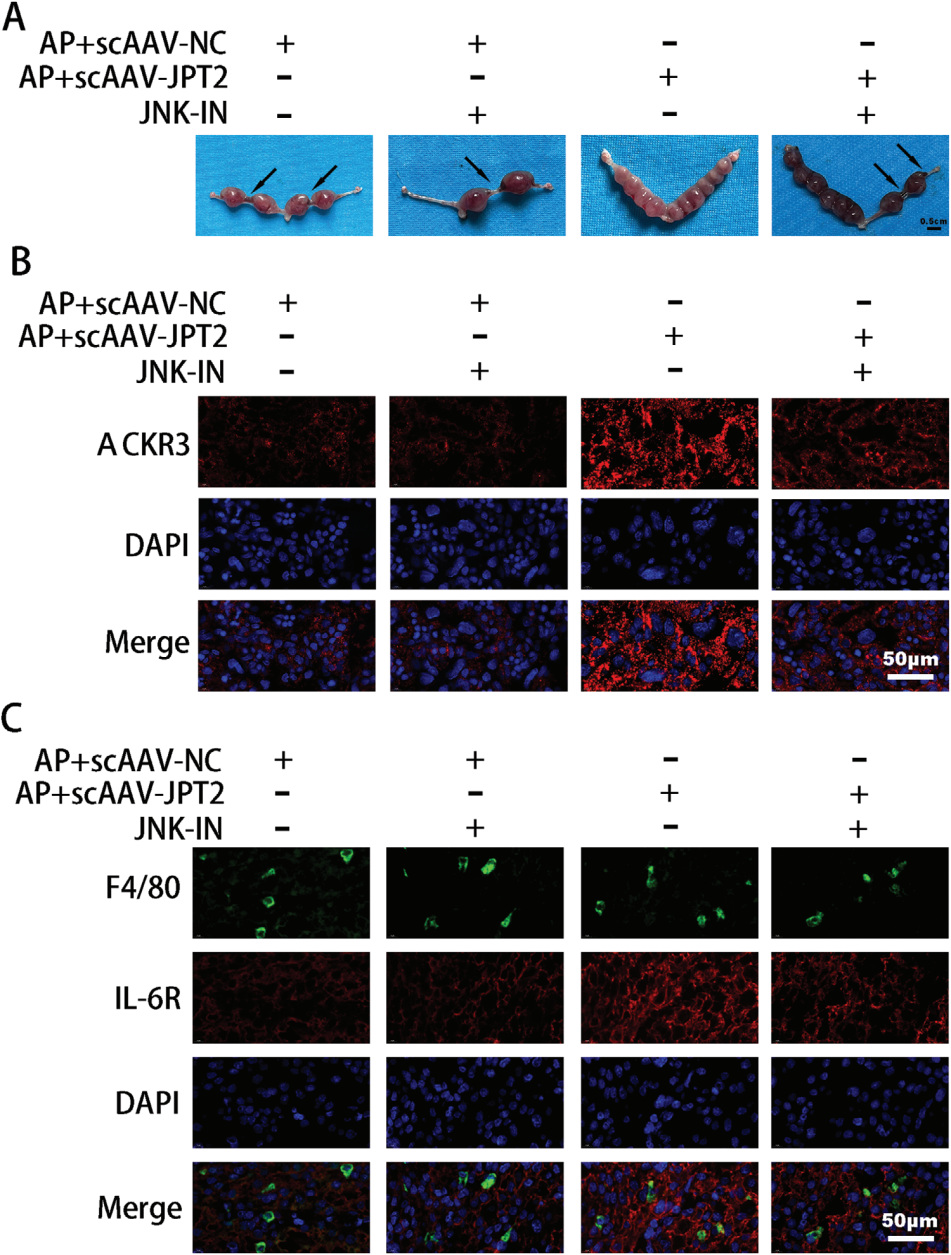
Activation of JNK signaling is vital for RSA treatment via JPT2. A) Pregnant mice received JNK inhibitor (JNK‐IN‐8, 20 mg kg^−1^) injections on days 8.5 and 10.5 of gestation, mice were euthanized on day 11.5 of gestation, and embryo resorption rates were calculated (each group: *n* = 8). Scale bar, 0.5 cm. B) Immunostaining ACKR3 at the placental interface of mice (each group: *n* = 8). Scale bar, 50 µm. C) Immunostaining of IL‐6R in the decidual macrophages of mice (each group: *n* = 8). Scale bar, 50 µm.

Next, we verified the role of JNK signaling in JPT2 expression in mice with miscarriages. JNK‐IN was used to block the JNK signaling. As shown in **Figures** **8**A and S17A (Supporting Information), JNK‐IN‐8 (a JNK inhibitor) significantly reversed the therapeutic effect of scAAV‐JPT2 on the embryo resorption rate in the AP group. The fluorescence results of ACKR3 showed that JNK‐IN‐8 significantly reduced ACKR3 expression in the placentas of the AP group mice (Figure 8B and Figure S17B, Supporting Information). Dual immunofluorescence results of F4/80 and IL‐6R showed that JNK‐IN‐8 significantly decreased the percentage of IL‐6R‐positive macrophages in the placenta of scAAV‐JPT2–augmented AP mice (Figure 8C and Figure S17C, Supporting Information). These data suggest that the activation of JNK signaling is likely critical for JPT2 treatment of RSA.

## 3 Discussion

**Figure 9:**
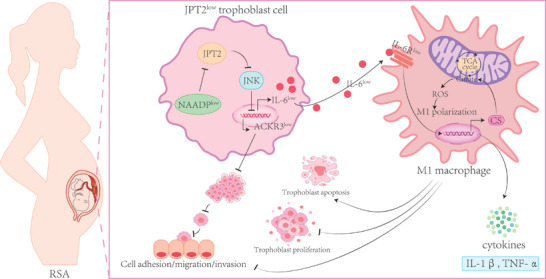
JPT2 deficiency inhibits trophoblast functions via the JNK signaling pathway and reprograms macrophage polarization, leading to RSA. On the one hand, JPT2 deficiency inhibited trophoblast adhesion, migration, and invasion by inhibiting the JNK/ACKR3 axis. On the other hand, the lack of JPT2 in trophoblasts contributed to M1 macrophage polarization by promoting the accumulation of citrate and ROS via inhibition of the JNK/IL‐6 axis, which subsequently affected the trophoblast functions. Cs: Citrate synthase.

RSA is a severe and challenging condition for parents hoping to have children.^[^ ^1^ ^]^ Approximately 50% of the patients with RSA exhibit an unknown etiology.^[^ ^7^ ^]^ Therefore, exploring the unknown causes and underlying mechanisms is important for RSA treatment. In this study, we identified, for the first time, the essential roles of JPT2 at the maternal–fetal interface. We found that JPT2 signaling was downregulated in both the villous tissue of patients with RSA and placentas of AP mice. Mechanistically, JPT2 deficiency inhibited trophoblast adhesion, migration, and invasion by inhibiting the JNK/ACKR3 axis. On the other hand, the lack of JPT2 in trophoblasts contributed to M1 macrophage polarization by promoting the accumulation of citrate and ROS via inhibition of the JNK/IL‐6 axis, which subsequently affected the trophoblast functions (**Figure** **9**). Therefore, abnormally low placental JPT2 levels may be an important cause of pregnancy‐related complications, including RSA.

JPT2 is expressed in various human tissues, such as the kidney, liver, and reproductive tissues.^[^ ^36^ ^]^ However, whether JPT2 is associated with placenta‐associated diseases, such as RSA, remains unknown. Here, we found that the levels of JPT2 were significantly downregulated in the villous tissues of patients with RSA and placentas of miscarried mice. Therefore, determination of JPT2 expression in the villous tissues of pregnant patients may aid in the clinical diagnosis of RSA. JPT2 is a highly sensitive NAADP‐binding protein that is essential for Ca^2+^ homeostasis.^[^ ^15^, ^37^ ^]^ Our data showed a significant decrease in calcium and NAADP concentrations in the placental villous tissue of patients with RSA and placentas of AP mice. LSM12 is another NAADP‐binding protein.^[^ ^15c^ ^]^ Here, LSM12 expression was not significantly altered in the villous tissues of patients with RSA or placentas of AP mice. In addition, we monitored the effect of general agonists (ATP and acetylcholine) on NAADP/JPT2‐mediated intracellular calcium mobilization in trophoblasts. Knockdown of JPT2 counteracted a portion of the ATP response to cytosolic calcium but not the acetylcholine response to cytosolic calcium. This may be due to the fact that ATP not only increases extracellular calcium inward flow but also increases calcium concentration by increasing intracellular NAADP concentration,^[^ ^38^ ^]^ whereas acetylcholine does not.^[^ ^39^ ^]^ Interestingly, NAADP treatment significantly reversed the decrease in calcium concentration in the placentas of miscarried mice. However, NAADP inhibition did not result in embryo resorption rates in NP mice similar to those observed in AP mice without Ned19. This suggests that the reduction of free calcium in the RSA placenta is related to NAADP/JPT2‐mediated calcium and that embryo loss is associated with the inhibition of NAADP signaling; however, additional potential mechanisms remain in RSA.

Recent studies on JPT2 have focused on cancer development and JPT2 is thought to promote tumor development. For example, it promotes the growth and metastasis of hepatocellular carcinoma tumors.^[^ ^14c^ ^]^ In addition, JPT2 is associated with the proliferation of non‐small cell lung cancers.^[^ ^14b^ ^]^ Zeng et al. demonstrated the important tumor‐promoting role of the JPT2/AP‐2γ/PLK1 signaling axis in esophageal cancer.^[^ ^14a^ ^]^ These results suggest that JPT2 plays an important role in regulating cell growth and migration. Interestingly, the proliferation, migration, and invasion abilities of placental trophoblasts are similar to those of tumor cells.^[^ ^40^ ^]^ However, the role of JPT2 in placental trophoblasts has not been investigated. Next, we explored the mechanisms mediated by JPT2 in trophoblasts. Combined with the RNA‐seq results, our study revealed that JPT2 regulates trophoblast adhesion, migration, and invasion. Unexpectedly, although TPCs are effectors of NAADP/JPT2, the absence of TPCs did not affect trophoblast adhesion, migration, or invasion, suggesting that JPT2 affects trophoblast function through other pathways. RNA‐seq results identified ACKR3 as the most significantly downregulated gene after JPT2 knockdown. ACKR3 is upregulated in many cancers and regulates cell survival and tumor growth.^[^ ^22^, ^41^ ^]^ Our findings demonstrate that, although there is no direct interaction between JPT2 and ACKR3, overexpression of ACKR3 can reverse trophoblast adhesion, migration, and invasion caused by JPT2 deficiency.

Fetal trophoblasts and maternal immune cells of the placenta interact at the maternal–fetal interface.^[^ ^42^ ^]^ Macrophages are important decidual immune cells essential for normal pregnancy and are involved in various pregnancy complications, including RSA.^[^ ^12^ ^]^ In our study, the number of M1‐type macrophages was higher in women with RSA than in HC, which is consistent with previous findings.^[^ ^43^ ^]^ However, many unknown factors are involved in M1/M2 macrophage homeostasis. Furthermore, we found that JPT2‐deficient trophoblasts promote M1 polarization and ROS accumulation in macrophages. Although we found that overexpression of ACKR3 affected the effect of JPT2 knockdown on trophoblast function, overexpression of ACKR3 did not affect macrophage M1 polarization, which was enhanced by JPT2 knockdown in trophoblasts. Trophoblasts can establish a mechanism regulating macrophage polarization at the maternal–fetal interface through secreted immune compounds.^[^ ^13b^ ^]^ In the present study, combined with the RNA‐seq results, we found that JPT2‐deficient trophoblasts promoted M1 polarization and ROS accumulation in macrophages, and this role may be exerted through the inhibition of IL‐6 secretion. Similarly, macrophages are crucial for regulating the biological behavior of trophoblasts.^[^ ^44^ ^]^ Our data showed that JPT2‐deficient trophoblast‐educated macrophages could inhibit trophoblast proliferation, adhesion, migration, and invasion, and promote trophoblast apoptosis.

Interestingly, we also observed an important role of JNK signaling in JPT2 regulation. Transcriptomics revealed that MAPK signaling was associated with JPT2 deficiency; therefore, we further analyzed the MAPK signaling pathway. MAPKs in mammals include JNK, p38 MAPK, and ERK, which are involved in various important cellular activities, including differentiation, growth, apoptosis, and inflammation.^[^ ^45^ ^]^ Here, we found that JPT2 was involved in the pathogenesis of RSA by regulating trophoblast function and macrophage polarization via JNK signaling, but not via p38 MAPK and ERK signaling.

Maintenance of the macrophage phenotype and function requires energy provided by cellular metabolism.^[^ ^30^, ^46^ ^]^ Metabolic and functional changes in decidual macrophages at the maternal–fetal interface may play important roles in the development of RSA. Our untargeted metabolomic data revealed that JPT2‐deficient trophoblasts enhanced citrate production in macrophages. Evidence suggests that citrate promotes ROS accumulation in macrophages.^[^ ^47^ ^]^ Accumulation of ROS promotes M1 polarization in macrophages.^[^ ^25^ ^]^ In addition, citrate accumulation contributes to the maintenance of M1 polarization in macrophages.^[^ ^32^ ^]^ Here, JPT2 deletion in trophoblasts inhibited IL‐6 expression, subsequently increasing the citrate levels and leading to the accumulation of ROS and M1 polarization in macrophages.

Finally, we administered scAAV‐JPT2 to NP and AP mice and found that overexpression of JPT2 in the placenta alleviated the embryo resorption rates, promoted trophoblast migration and invasion, and inhibited macrophage M1 polarization. Furthermore, although NAADP supplementation alleviated the embryo resorption rates to some extent, scAAV‐JPT2, which targets JPT2, was significantly more effective than NAADP. These findings suggest the potential of JPT2‐targeted strategies for the clinical treatment of RSA. However, although we used an adeno‐associated virus to promote JPT2 expression in mice, its effect was not limited to trophoblasts and was also affects other cells, including macrophages and mesenchymal cells, which is a limitation of this study. We hope to address this issue in our future investigations.

In summary, we found that JPT2 expression at the maternal–fetal interface was a key regulator of trophoblast functions and macrophage metabolic homeostasis. Moreover, JPT2 expression at the maternal–fetal interface regulated the trophoblast–macrophage crosstalk. Overall, our findings suggest JPT2 as a prospective target for RSA therapy.

## 4 Experimental Section

The materials and methods used in this study have been described in detail in the Supporting Information.

## Conflict of Interest

The authors declare no conflict of interest.

## Author Contributions

X.C. and Q.L.S. equally contributed to this study. X.C. and Q.L.S. are first co‐authors. X.C. and Q.L.S. designed the experiments. R.J., J.Y.W., and M.L.C. collected the specimens and performed the follow‐up. D.Y.G., Y.Z., and J.Y. conducted the data analysis and interpretation. X.C., Q.L.S., D.Y.G., Y.Z., and J.Y. wrote the manuscript. All the authors have read and approved the final version of the manuscript.

## Supporting information

Supporting Information

## Data Availability

The data that support the findings of this study are available from the corresponding author upon reasonable request.
