# Supplementary material for: JPT2 Affects Trophoblast Functions and Macrophage Polarization and Metabolism, and Acts as a Potential Therapeutic Target for Recurrent Spontaneous Abortion
Source: Adv Sci (Weinh). 2024 Feb 28;11(16):2306359. doi: 10.1002/advs.202306359 (PMC11040346; doi:10.1002/advs.202306359)
Supplement: Supplementary file 1 — Supporting Information [file ADVS-11-2306359-s001.pdf]

ADVANCED SCIENCE
Open Access

Supporting Information

for *Adv. Sci.*, DOI 10.1002/advs.202306359

JPT2 Affects Trophoblast Functions and Macrophage Polarization and Metabolism, and Acts as a Potential Therapeutic Target for Recurrent Spontaneous Abortion

Xin Chen, Qian Lin Song, Rui Ji, Jia Yu Wang, Ming Liang Cao, Duan Ying Guo, Yan Zhang* and Jing Yang**

Supporting Information 1

JPT2 affects trophoblast functions and macrophage polarization and metabolism and acts as a potential therapeutic target for recurrent spontaneous abortion

Xin Chen[1], Qian Lin Song[2], Rui Ji[1], Jia Yu Wang[1], Ming Liang Cao[3], Duan Ying Guo[4, *], Yan Zhang[3, *], Jing Yang[1, *]

Supplementary figures

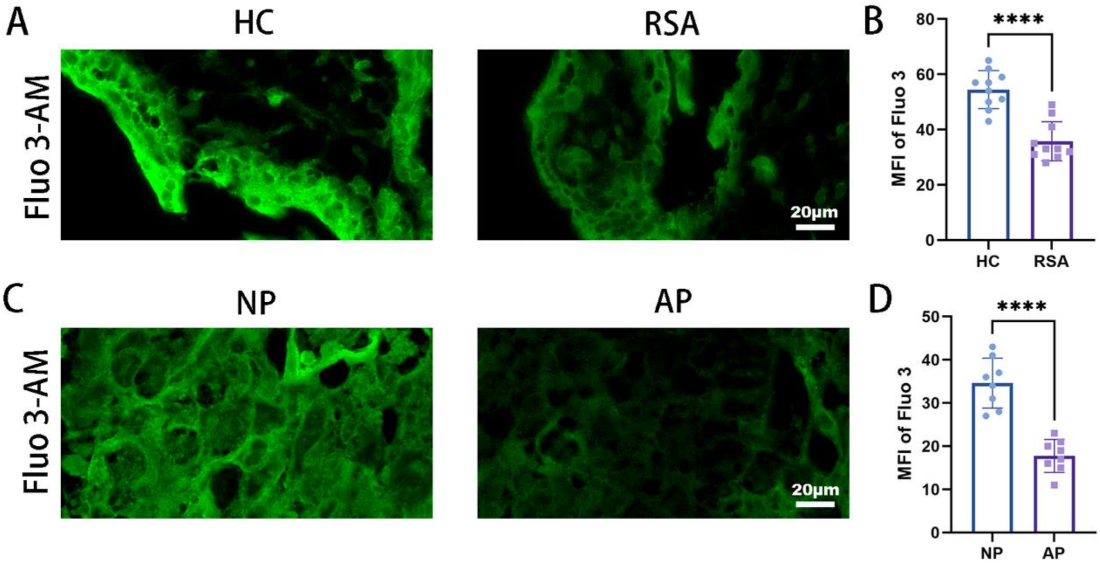

Figure S1. The calcium content in the villous tissue of RSA patients and the placenta of miscarriage mice is reduced. (A-B) The calcium concentration in the villous tissue of the normal pregnancy group (healthy control, HC) and RSA group was measured by Fluo 3-AM (each group: n=10). (A)Representative fluorescence images of Fluo3-AM in the villous tissue. Scale bar, 20 μm. (B)Quantitative values of Fluo 3 mean fluorescence intensity (MFI) (each group: n=10). **(C-D)** The calcium concentration in the placental tissues of mice in the NP group and AP group was measured by Fluo 3-AM (each group: n=8). (C)Representative fluorescence images of Fluo 3-AM at the mouse placental interface. Scale bar, 20 μm. (D)Quantitative values of Fluo 3 MFI (each group: n=8). Error bars indicate the SD of the mean. Student's t-test was used to assess differences between the two groups. ****$P < 0.0001$.

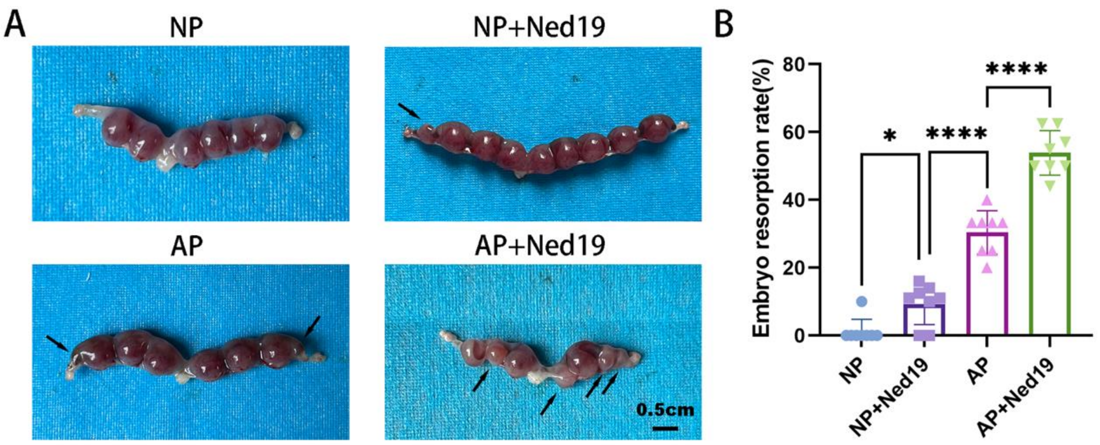

Figure S2. The embryo loss in mice is associated with inhibition of NAADP signaling. NP and AP mice were intervened with an NAADP inhibitor (Ned19, 20 mg/kg), and mice were euthanized on day 11.5 of gestation (each group: n=8). **(A)** The black arrow points to the resorption of the embryo. Scale bar, 0.5cm. **(B)** Determination of embryo resorption rates at gestation day 11.5 (each group: n=8). Error bars indicate the SD of the mean. One-way ANOVA was used to compare differences between multiple groups. *P <0.05, ****P < 0.0001.

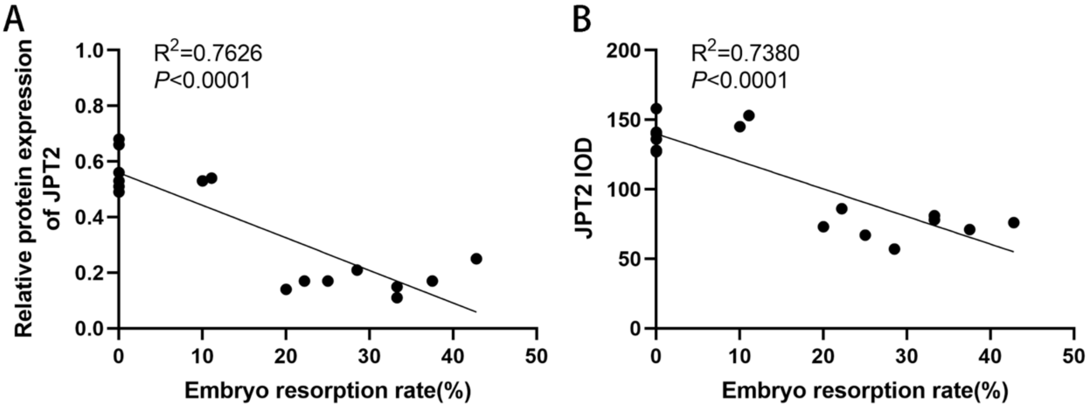

Figure S3. The expression of JPT2 in the mice placenta is negatively correlated with the embryo resorption rate. (A) The associations between embryo resorption rate and JPT2 protein expression were investigated through Pearson correlation analysis. **(B)** The associations between embryo resorption rate and JPT2 IOD were investigated through Pearson correlation analysis. Error bars indicate the SD of the mean. *P < 0.05.

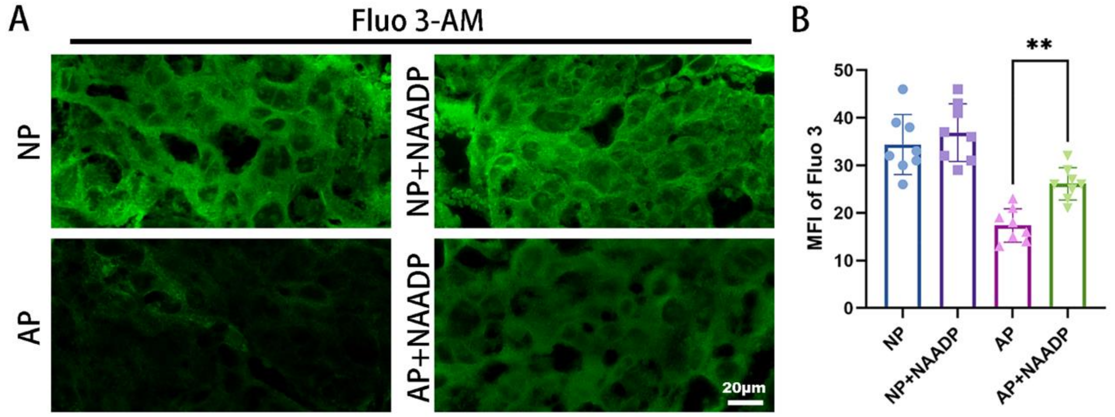

Figure S4. NAADP restores the free calcium content in the placenta of AP mice. (A-B) Pregnant mice were randomly divided into 4 groups (NP, NP+NAADP (0.181 mg/kg), AP, and AP+NAADP (0.181 mg/kg) groups) (each group: n=8) and subsequently assayed for placental calcium concentration by Fluo 3-AM. (A)Representative fluorescence images of Fluo 3-AM at the mouse placental interface. Scale bar, 20 µm. (B)Quantitative values of Fluo 3 MFI (each group: n=8). Error bars indicate the SD of the mean. One-way ANOVA was used to compare differences between multiple groups. **P <0.01.

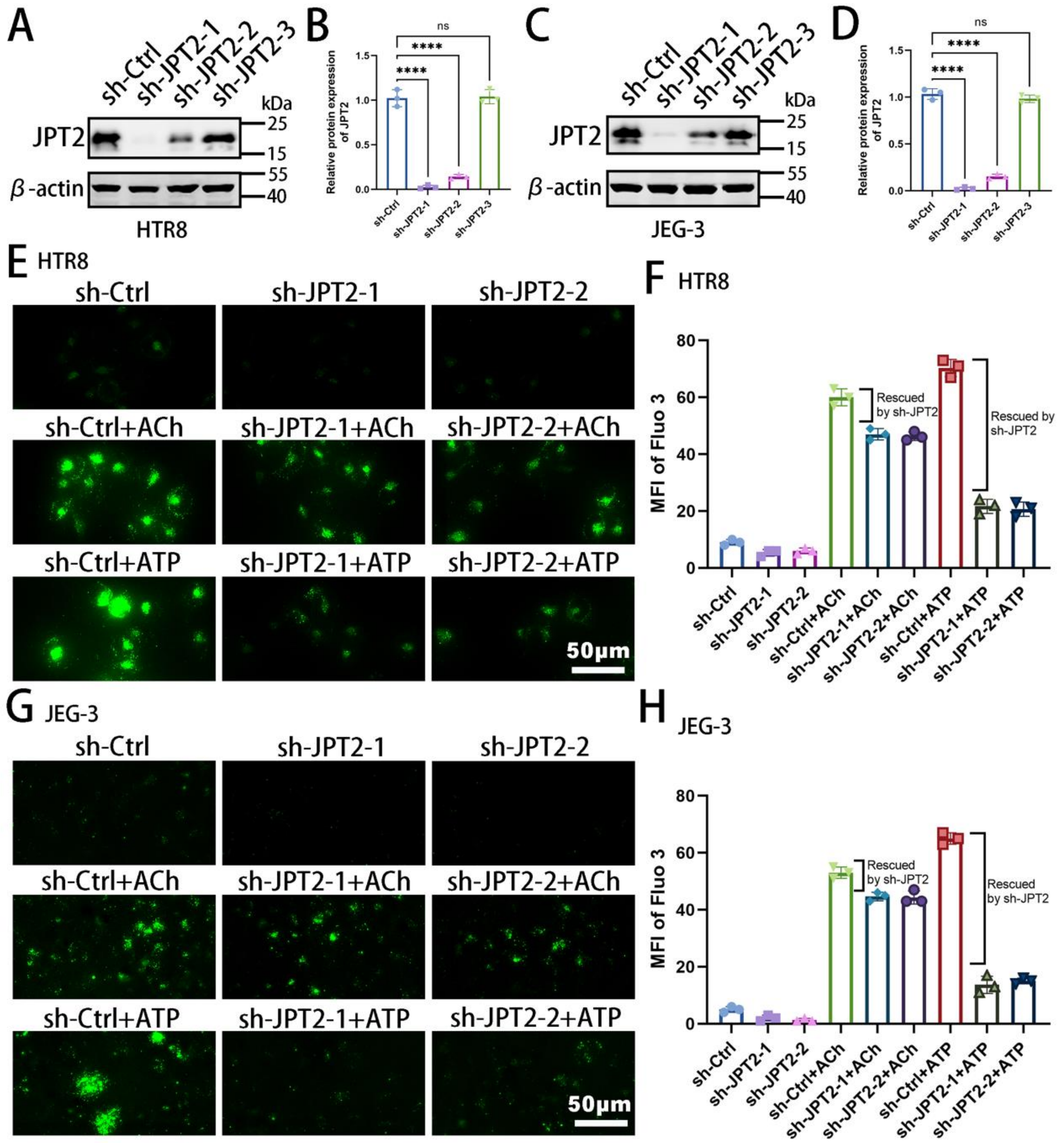

Figure S5. Knockdown of JPT2 inhibits the response of ATP to calcium to a certain extent but does little to alter the response of acetylcholine to calcium. (A-D) HTR8 and JEG-3 cells were infected with JPT2 knockdown lentiviral vectors and virus-negative controls, and JPT2 expression was assessed by western blotting. **(E and G)** Representative fluorescence images of Fluo 3-AM in HTR8 and JEG-3 cells. Scale bar, 50 μm. **(F and H)** Quantitative values of Fluo 3 MFI. Data represent mean ± SD of at least three independent experiments, with each data point representing an independent experiment. Error bars indicate the SD of the mean. One-way ANOVA was used to compare differences between multiple groups. ****P < 0.0001, ns: not significant.

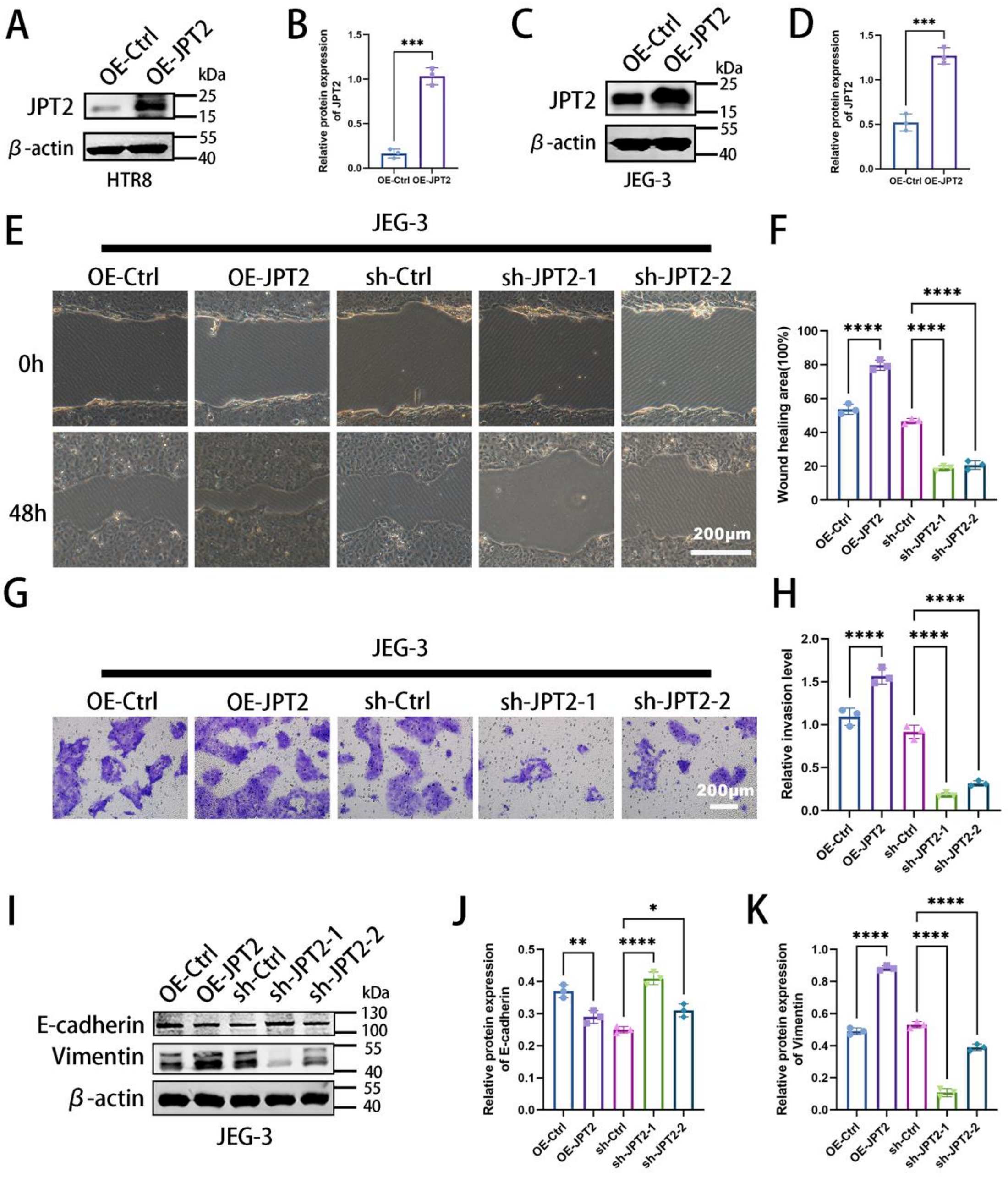

Figure S6. JPT2 regulates the migration and invasion of trophoblast cells. (A-D) HTR8 and JEG-3 cells were infected with JPT2 overexpressing lentiviral vectors and negative viral controls, and JPT2 expression was assessed by western blotting. **(E-F)** Quantitative values of migration ability and migration ability of JEG-3 cells were detected by wound healing assays. Scale bar, 200 μm. **(G-H)** Quantitative values of invasive ability and invasive capacity of JEG-3 cells detected by transwell assays. Scale bar, 200 μm. **(I-K)** Protein blot images and quantitative values of E-cadherin

and Vimentin protein levels in JPT2 knockdown or overexpressed JEG-3 cells. (I) Representative protein blot images. (J) Quantification values of E-cadherin protein levels. (K) Quantitative values of Vimentin protein levels. Data represent mean ± SD of at least three independent experiments, with each data point representing an independent experiment. Error bars indicate the SD of the mean. Student's t-test was used to assess differences between the two groups, and one-way ANOVA was used to compare differences between multiple groups. *P < 0.05, **P < 0.01, ***P < 0.001, ****P < 0.0001.

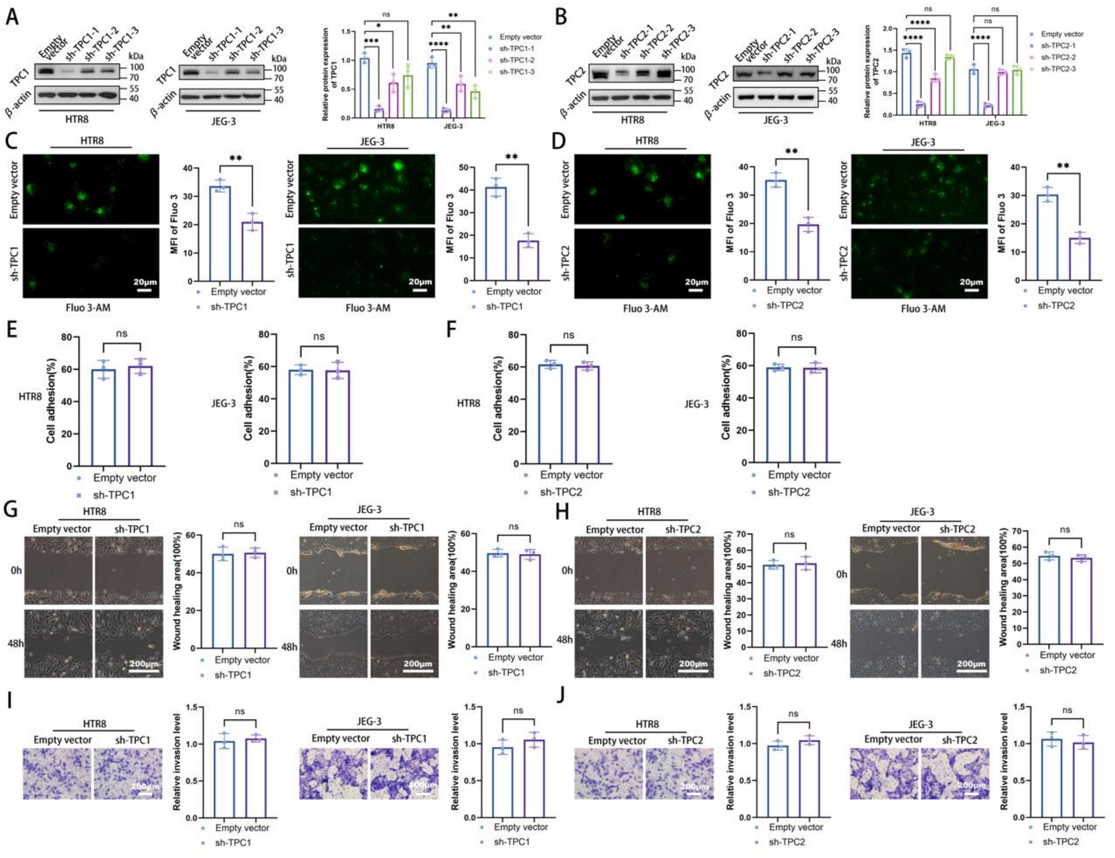

Figure S7 JPT2 affects intracellular calcium changes through TPCs but does not affect trophoblast cell behavior through TPCs. (A-B) After the intervention of HTR8 and JEG-3 cells with empty vector plasmid and TPC1 or TPC2 knockdown plasmid, the expression of TPC1 or TPC2 was evaluated by Western blotting. **(C-D)** Representative fluorescence images of Fluo 3-AM

and quantitative values of Fluo 3 MFI in HTR8 and JEG-3 cells. Scale bar, 20 μm. **(E-F)** The cell adhesion ability of TPC1 or TPC2 knockdown HTR8 and JEG-3 cells. **(G -H)** Migration ability and quantification values of HTR8 and JEG-3 cells were examined by wound healing assay. Scale bar, 200 μm. **(I - J)** The invasive ability and quantitative values of HTR8 and JEG-3 cells were detected by transwell assays. Scale bar, 200 μm. Data represent mean ± SD of at least three independent experiments, with each data point representing an independent experiment. Error bars indicate the SD of the mean. Student's t-test was used to assess differences between the two groups, and one-way ANOVA was used to compare differences between multiple groups. *P < 0.05, **P < 0.01, ***P < 0.001, ****P < 0.0001, ns: not significant.

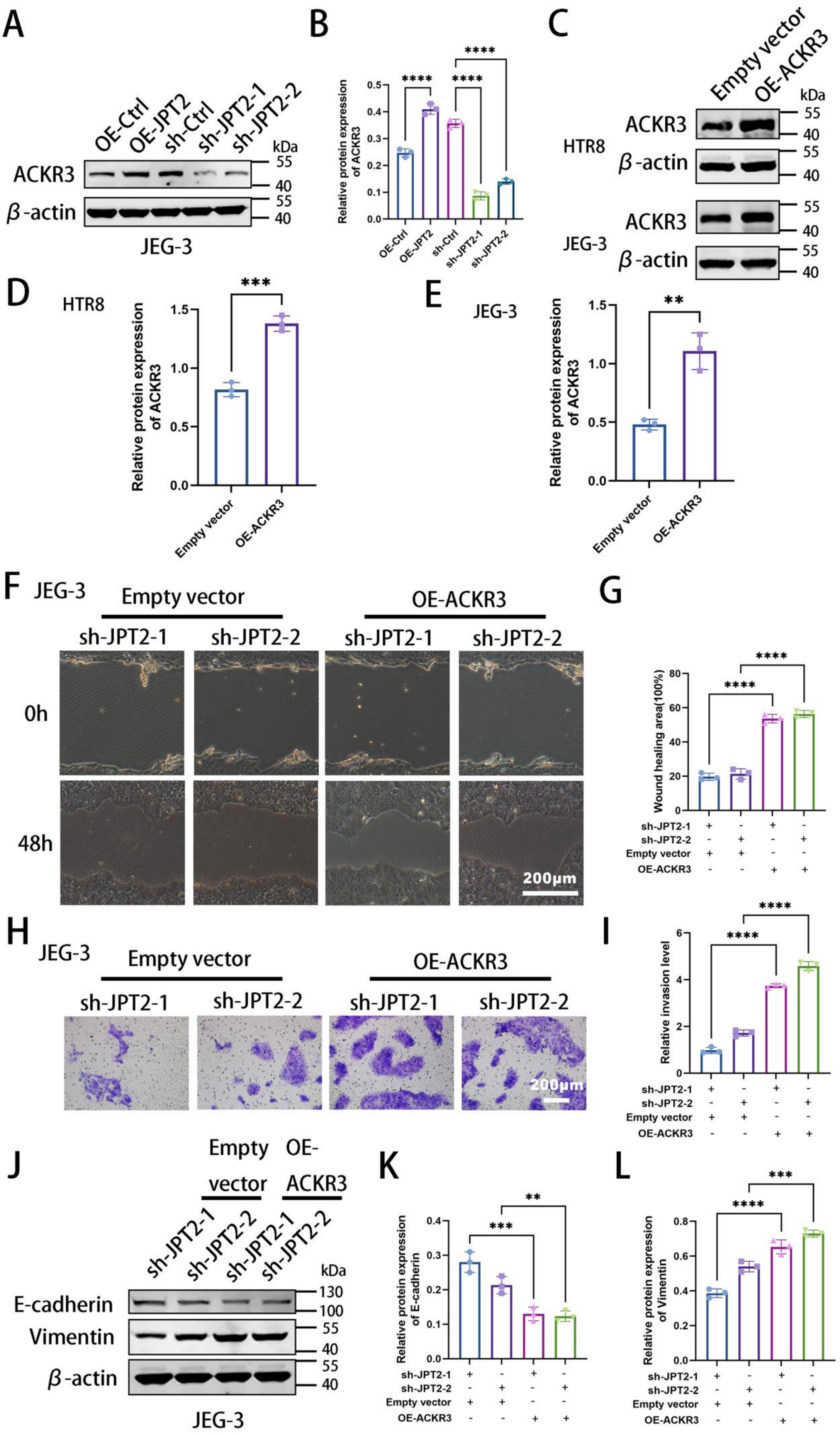
A
OE-Ctrl
OE-JPT2
sh-Ctrl
sh-JPT2-1
sh-JPT2-2
kDa
ACKR3
β-actin
55
40
JEG-3
B
Relative protein expression of ACKR3
****
OE-Ctrl
OE-JPT2
sh-Ctrl
sh-JPT2-1
sh-JPT2-2
C
Empty vector
OE-ACKR3
HTR8
JEG-3
D
HTR8
Relative protein expression of ACKR3
***
E
JEG-3
**
F
JEG-3
Empty vector
OE-ACKR3
sh-JPT2-1
sh-JPT2-2
0h
48h
200μm
G
Wound healing area(100%)
Empty vector
OE-ACKR3
H
I
Relative invasion level
J
Empty vector
OE-ACKR3
E-cadherin
Vimentin
130
100
K
Relative protein expression of E-cadherin
L
Relative protein expression of Vimentin

Figure S8. ACKR3 is critical for JPT2 to regulate trophoblast function. (A-B) Protein blot images and quantitative values of ACKR3 protein levels in JPT2 knockdown or overexpressed JEG-3 cells. **(C-E)** After the intervention of HTR8 and JEG-3 cells with empty vector plasmid and ACKR3 overexpression plasmid, the expression of ACKR3 was evaluated by Western blotting. (C) Representative protein blot images. (D-E) Quantitative values of ACKR3 protein levels in HTR8 and JEG-3 cells. **(F-I)** Effect of ACKR3 overexpression on trophoblast migration and invasion. (F-G) Images and quantified values of Migration ability of JEG-3 cells. Scale bar, 200 μm. (H-I) Images and quantified values of invasive ability of JEG-3 cells. Scale bar, 200 μm. **(J)** Representative protein blot images of E-cadherin and Vimentin protein levels in JEG-3 cells. **(K)** Quantitative values of E-cadherin protein levels. **(L)** Quantitative values of Vimentin protein levels. Data represent mean ± SD of at least three independent experiments, with each data point representing an independent experiment. Error bars indicate the SD of the mean. Student's t-test was used to assess differences between the two groups, and one-way ANOVA was used to compare differences between multiple groups. $^{**}P < 0.01$, $^{***}P < 0.001$, $^{****}P < 0.0001$.

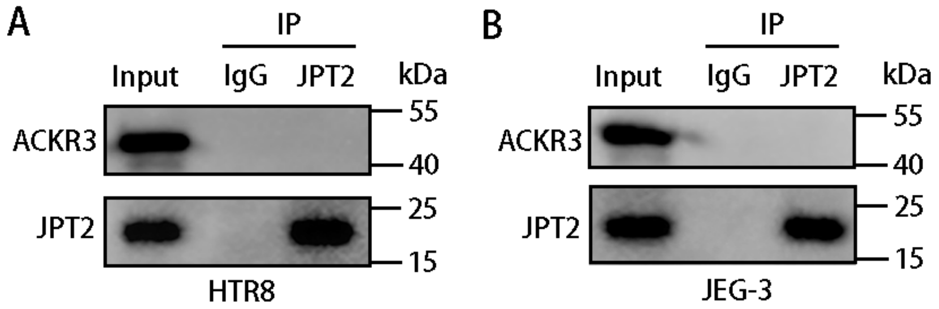

Figure S9. JPT2 and ACKR3 have no direct interaction. (A-B) Representative Co-immunoprecipitation images. Data represent mean ± SD of at least three independent experiments.

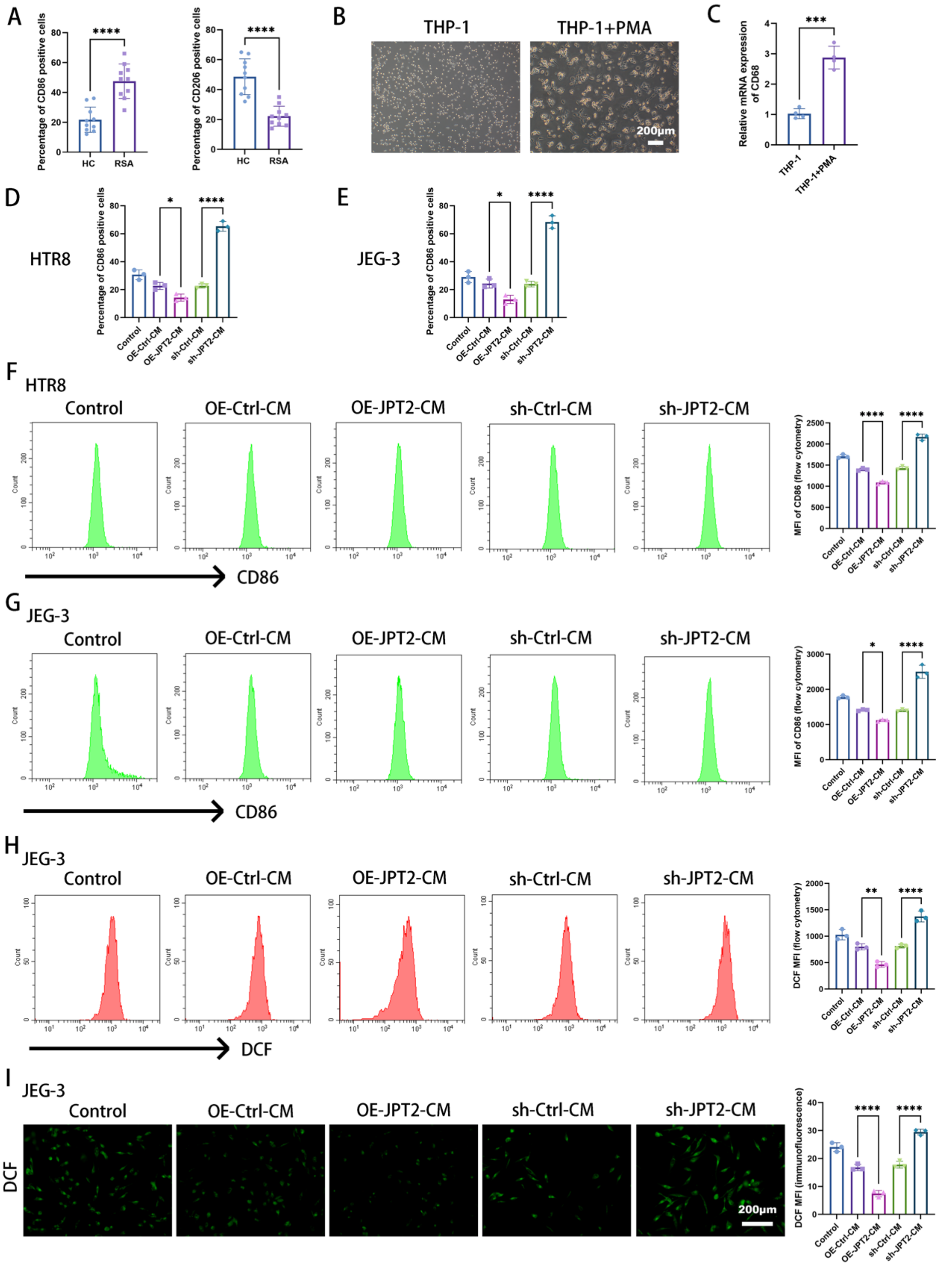

Figure S10. JPT2-deficient trophoblasts promote M1 polarization and the accumulation of ROS in macrophages. (A) The percentage of CD86 and CD206 positive cells in decidual macrophages of women with HC and RSA (each group: n=10). **(B)** Macrophages derived from THP1 induced using PMA (100 ng/mL) for 24 h were assessed by morphology. Scale bar, 200 μm.

(C) CD68 mRNA expression was used to identify the macrophages derived in Figure S10B. (**D-E**) The percentage of CD86 and CD206 positive cells in macrophages treated with conditioned medium of HTR8 and JEG-3 cells. **(F-G)** The mean fluorescence intensity (MFI) of CD86 and quantification values were examined by flow cytometry in macrophages treated with a conditioned medium of HTR8 and JEG-3 cells. (**H-I**) Flow cytometry and representative fluorescence images showing the fluorescence intensity of DCF in macrophages treated with conditioned medium of JEG-3 cells. Scale bar, 200 μm. Data represent mean ± SD of at least three independent experiments, with each data point representing an independent experiment. Error bars indicate the SD of the mean. Student's t-test was used to assess differences between the two groups, and one-way ANOVA was used to compare differences between multiple groups. *$P < 0.05$, **$P < 0.01$, ***$P < 0.001$, ****$P < 0.0001$.

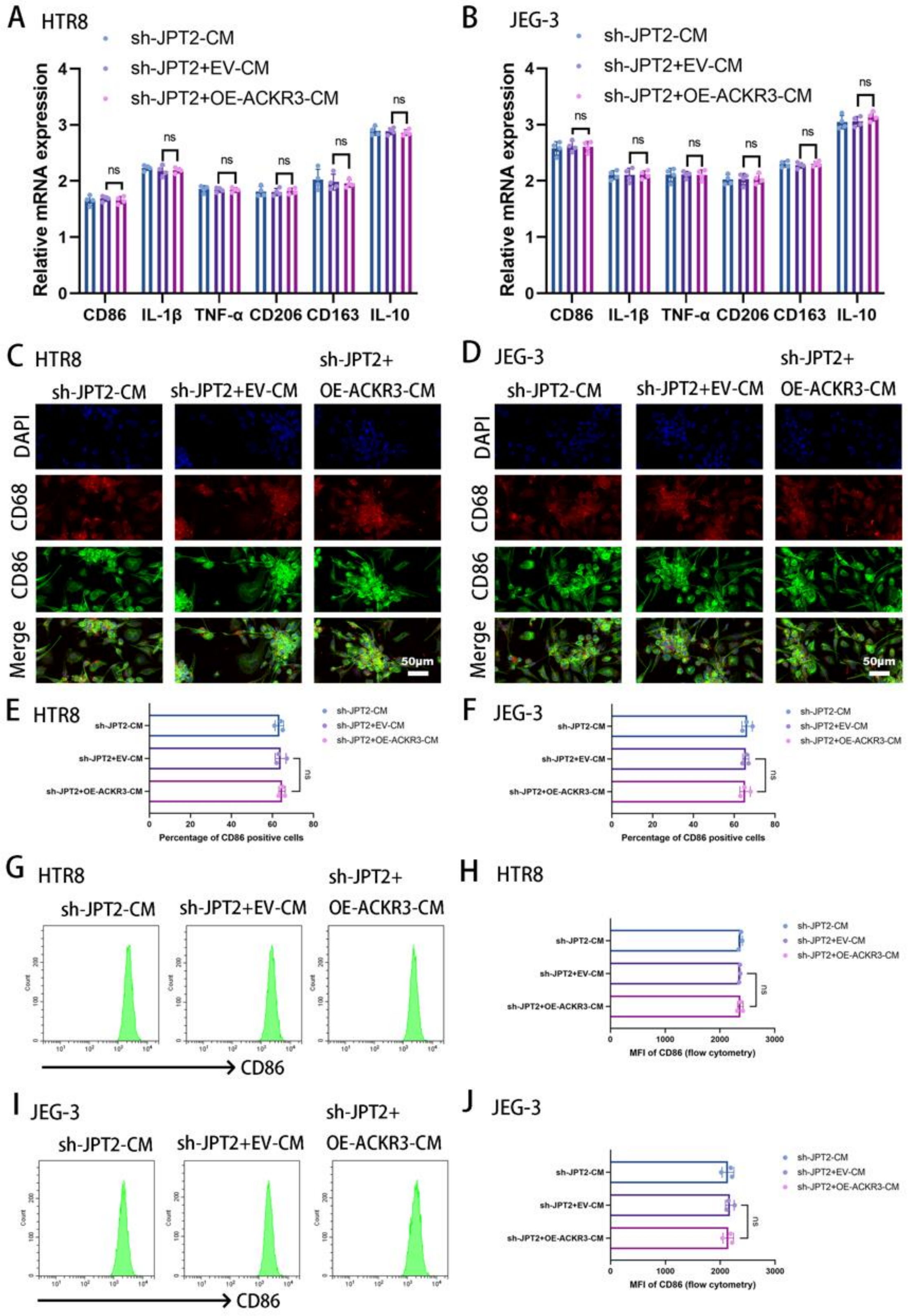

Figure S11. JPT2 changes in trophoblasts do not affect macrophage polarization through ACKR3. Knocked down JPT2 and overexpressed ACKR3 genes in HTR8 and JEG-3 cells, and monitored the macrophage polarization status after culturing the macrophages using the

trophoblastic conditioned medium. **(A-B)** The mRNA expression levels of M1 macrophage markers (CD86, IL-1β, TNF-α) and M2 macrophage markers (CD206, CD163, IL10) in macrophages after intervention with conditioned medium derived from HTR8 and JEG-3 cells were measured by qPCR. **(C-D)** Representative fluorescence images of CD68 and CD86 in macrophages treated with conditioned medium of HTR8 and JEG-3 cells. Scale bar, 50 μm. **(E-F)** Quantitative values of percentage of CD86 positive cells. **(G -J)** MFI of CD86 and quantification values were examined by flow cytometry in macrophages treated with a conditioned medium of HTR8 and JEG-3 cells. Data represent mean ± SD of at least three independent experiments, with each data point representing an independent experiment. Error bars indicate the SD of the mean. One-way ANOVA was used to compare differences between multiple groups. ns: not significant. EV: empty vector.

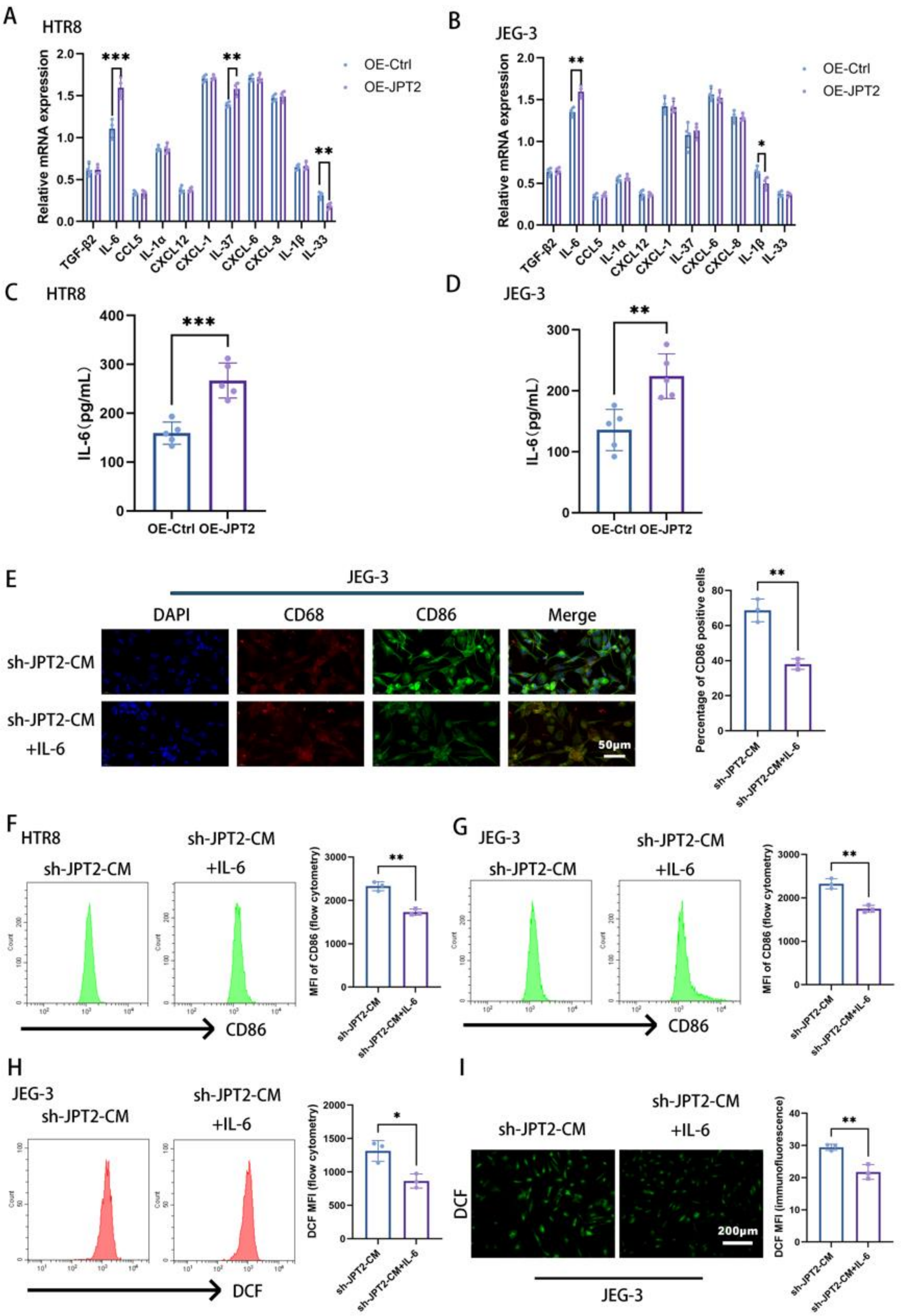

Figure S12. Deficiency of JPT2 in trophoblast cells promotes M1 polarization and ROS accumulation in macrophages by inhibiting IL-6 secretion. (A-B) Differential expression of selected cytokines and chemokines from Figure 3H in JPT2 overexpressing HTR8 and JEG-3 cells

was detected by qPCR. **(C-D)** Expression of IL-6 in the supernatant of JPT2 overexpressing HTR8 and JEG-3 cells was detected by ELISA. **(E)** Representative fluorescent images of CD68 and M1 macrophage marker CD86 and quantitative values of percentage of CD86 positive cells after treatment of macrophages using supernatants of JEG-3 cells with or without IL-6 (50ng/mL). Scale bar, 50 μm. **(F-G)** MFI of CD86 and quantification values were examined by flow cytometry in macrophages treated with a conditioned medium of HTR8 and JEG-3 cells with or without IL-6 (50ng/mL). **(H)** Detection of DCF MFI in macrophages treated with JEG-3 cell-conditioned medium with or without IL-6 (50ng/mL) by flow cytometry. **(I)** Representative fluorescence images and quantification of DCF fluorescence in macrophages treated with JEG-3 cell-conditioned medium with or without IL-6 (50ng/mL). Scale bar, 200 μm. Data represent mean ± SD of at least three independent experiments, with each data point representing an independent experiment. Error bars indicate the SD of the mean. Student's t-test was used to assess differences between the two groups. $^*P < 0.05$, $^{**}P < 0.01$, $^{***}P < 0.001$.

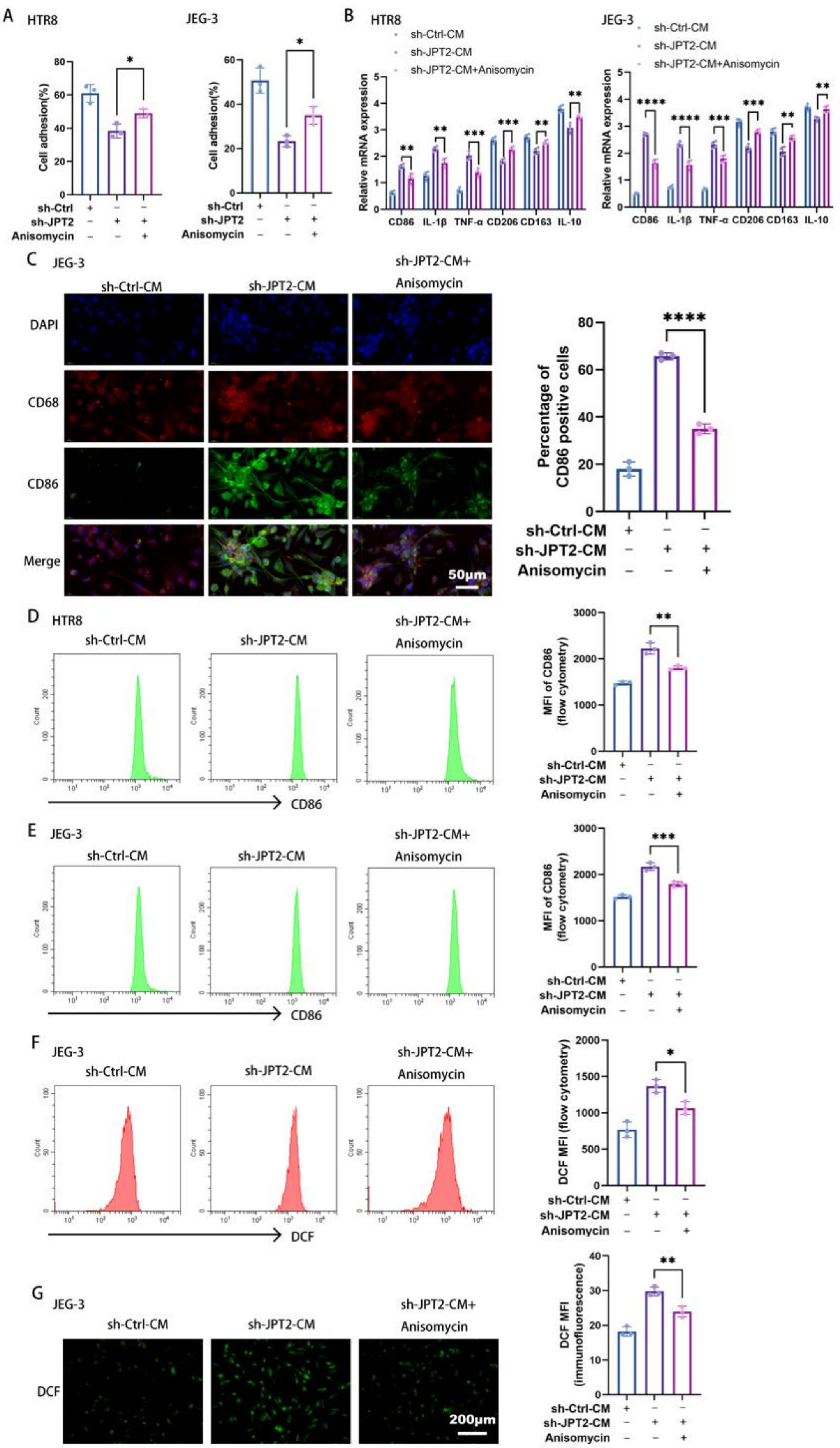

A
HTR8
JEG-3
Cell adhesion(%)
sh-Ctrl
sh-JPT2
Anisomycin
B
HTR8
JEG-3
sh-Ctrl-CM
sh-JPT2-CM
sh-JPT2-CM+Anisomycin
Relative mRNA expression
CD86
IL-1β
TNF-α
CD206
CD163
IL-10
C
JEG-3
sh-Ctrl-CM
sh-JPT2-CM
sh-JPT2-CM+ Anisomycin
DAPI
CD68
CD86
Merge
50μm
Percentage of CD86 positive cells
sh-Ctrl-CM
sh-JPT2-CM
Anisomycin
D
HTR8
Count
CD86
MFI of CD86 (flow cytometry)
E
JEG-3
F
JEG-3
DCF
DCF MFI (flow cytometry)
G
JEG-3
DCF
200μm
DCF MFI (immunofluorescence)

Figure S13. JNK signaling is critical for JPT2 to regulate trophoblast function and M1 polarization in macrophages. (A) Changes in trophoblast adhesion capacity after treatment with JNK activator Anisomycin (10 μM). **(B-G)** After pretreatment of trophoblast cells with JNK activator Anisomycin (10 μM), a conditioned medium of trophoblast cells was used to interfere with macrophages. (B)The mRNA expression levels of M1 macrophage markers (CD86, IL-1β, TNF-α) and M2 macrophage markers (CD206, CD163, IL-10) were detected in macrophages by qPCR. (C)Representative fluorescent images of CD68 and CD86 in macrophages and quantitative values of percentage of CD86 positive cells. Scale bar, 50 μm. (D-E) MFI of CD86 and quantification values were examined by flow cytometry in macrophages treated with a conditioned medium of HTR8 and JEG-3 cells. (F) Detection of DCF fluorescence and quantification of DCF fluorescence in macrophages treated with JEG-3 cell-conditioned medium by flow cytometry. (G) Representative fluorescence images of DCF and quantification of DCF MFI in macrophages treated with JEG-3 cell conditioned medium. Scale bar, 200 μm. Data represent mean ± SD of at least three independent experiments, with each data point representing an independent experiment. Error bars indicate the SD of the mean. One-way ANOVA was used to compare differences between multiple groups. *P < 0.05, **P < 0.01, ***P < 0.001, ****P < 0.0001.

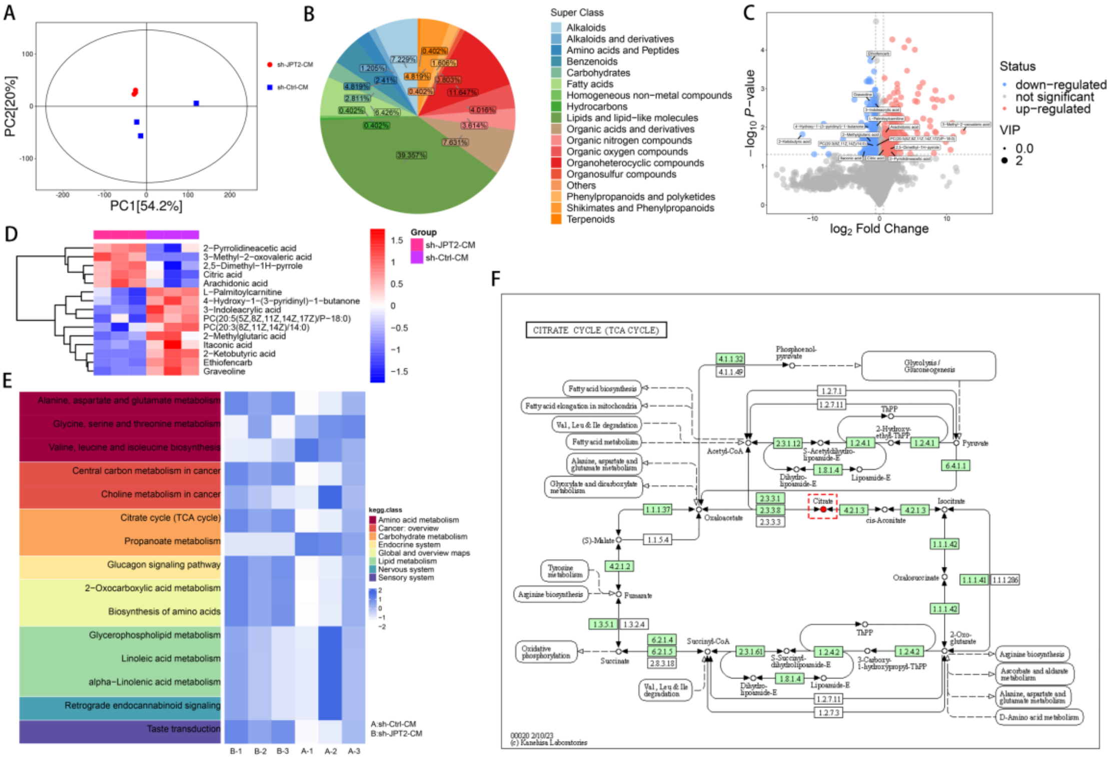

Figure S14. JPT2-deficient trophoblasts enhance citrate production in macrophages (A) Scatter plot of principal component analysis (PCA) scores for differential metabolites. **(B)** Distribution of the types of differential metabolites in macrophages after treatment with conditioned medium from JPT2 knockout and control groups of HTR8 cells. **(C)** Volcano plot showing the differential metabolites between the two groups. **(D)** Heatmap showing the differential expression of the differential metabolites labeled in Figure S14C. **(E)** Metabolic pathway maps for differential metabolite enrichment. **(F)** Flow diagram of metabolite changes in the TCA cycle pathway. Data represent mean ± SD of at least three independent experiments. Error bars indicate the SD of the mean.

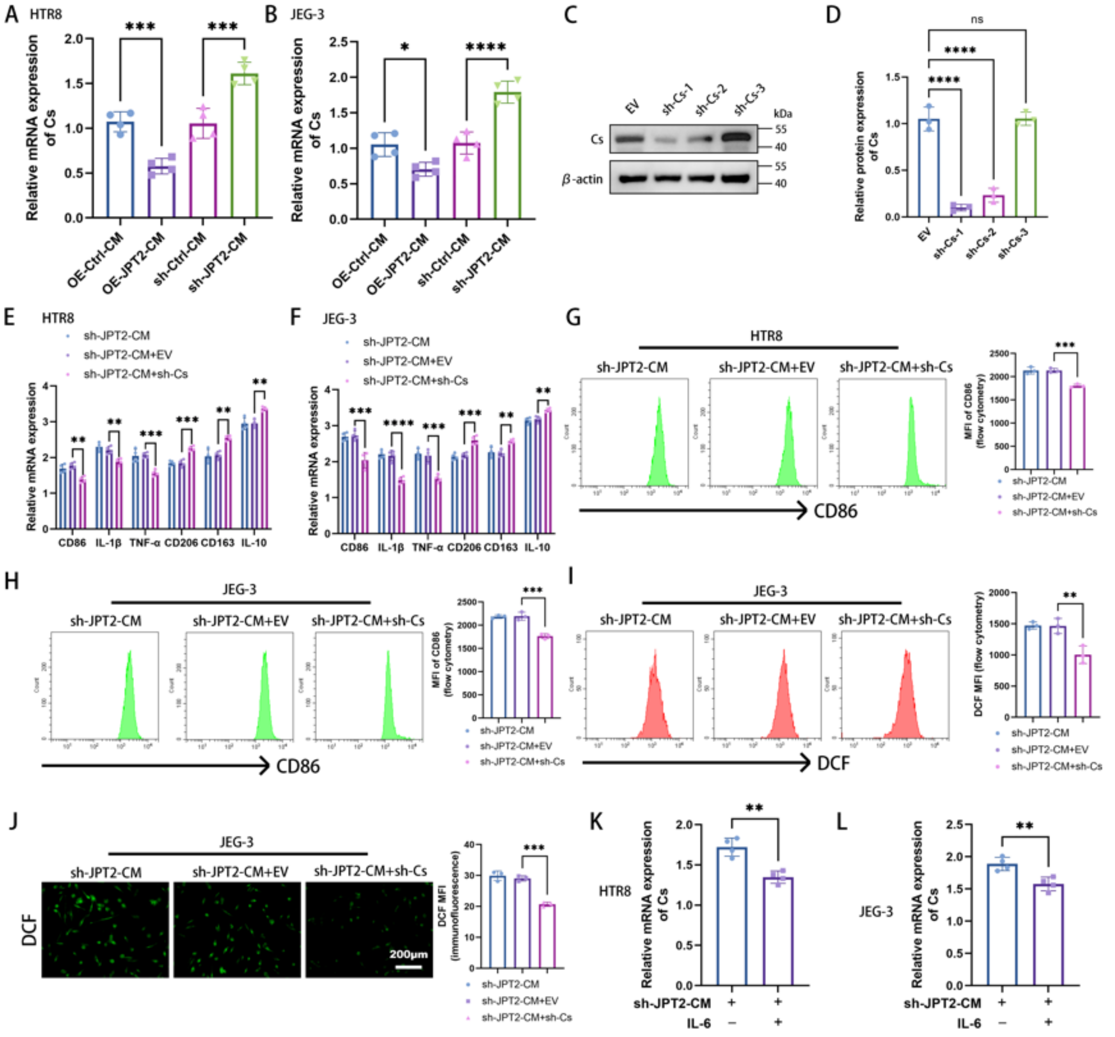

Figure S15. JPT2-deficient trophoblasts promote M1 polarization and the accumulation of ROS in macrophages by enhancing citrate production in macrophages. (A-B) The mRNA expression of Citrate synthase (Cs) in macrophages was detected using qPCR after intervention with a conditioned medium of HTR8 and JEG-3 cells. **(C-D)** After treating macrophages with empty vector (EV) plasmids and sh-Cs plasmids, Cs expression was assessed by western blotting. **(E-F)** After treating macrophages with empty vector plasmids and sh-Cs plasmids, macrophages were intervened with culture supernatants of HTR8 and JEG-3 cells. Then, the mRNA expression levels of M1 macrophage markers (CD86, IL-1β, TNF-α) and M2 macrophage markers (CD206, CD163, IL10) were detected in macrophages by qPCR. **(G-H)** MFI of CD86 and quantification values were

examined by flow cytometry in macrophages treated with a conditioned medium of HTR8 and JEG-3 cells. **(I)** DCF fluorescence and DCF fluorescence quantification were detected by flow cytometry in macrophages treated with conditioned medium from JEG-3 cells. **(J)** Representative fluorescence images of DCF and quantification of DCF MFI in macrophages treated with conditioned medium of JEG-3 cells. Scale bar, 200 μm. **(K-L)** Intervention of macrophages with conditioned medium from HTR8 and JEG-3 cells with or without IL-6 (50ng/mL). The mRNA expression levels of Cs from macrophages were detected by qPCR. Data represent mean ± SD of at least three independent experiments, with each data point representing an independent experiment. Error bars indicate the SD of the mean. Student's t-test was used to assess differences between the two groups, and one-way ANOVA was used to compare differences between multiple groups. *P < 0.05, **P < 0.01, ***P < 0.001, ****P < 0.0001, ns: not significant.

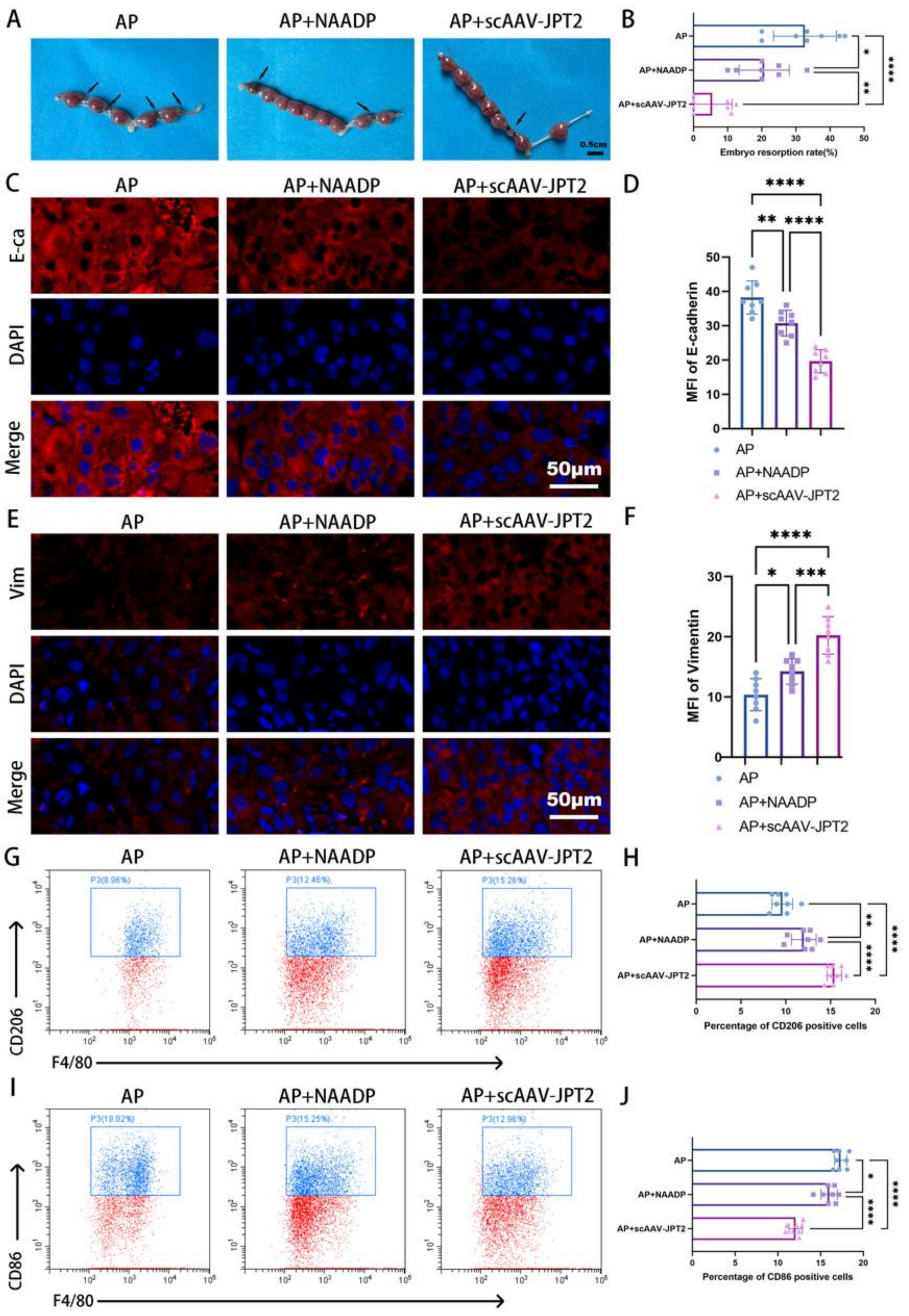

Figure S16. The therapeutic effect of NAADP is insufficient compared to scAAV-JPT2. Pregnant mice were randomly divided into 3 groups (AP, AP+NAADP (0.181 mg/kg), and AP+scAAV-JPT2 group), and embryo resorption rates of each group of pregnant mice were

measured at day 11.5 of gestation (each group: n=8). **(A)** Black arrows point to embryo resorption. Scale bar, 0.5cm. **(B)** Statistical results of embryo resorption (each group: n=8)**. (C)** Representative fluorescence images of E-cadherin (E-cad) at the mouse placental interface. Scale bar, 50 μm. **(D)** Quantitative values of E-cadherin mean fluorescence intensity (MFI) (each group: n=8). **(E-F)** Representative fluorescence images and quantified values of Vimentin (Vim) at the placental interface of mice (each group: n=8). Scale bar, 50 μm. **(G-H)** Representative flow cytometry results of CD206 in decidua macrophages of mice (each group: n=8). **(I-J)** Representative flow cytometry results of CD86 in decidua macrophages of mice (each group: n=8). Error bars indicate the SD of the mean. One-way ANOVA was used to compare differences between multiple groups. *P < 0.05, **P < 0.01, ***P < 0.001, ****P < 0.0001.

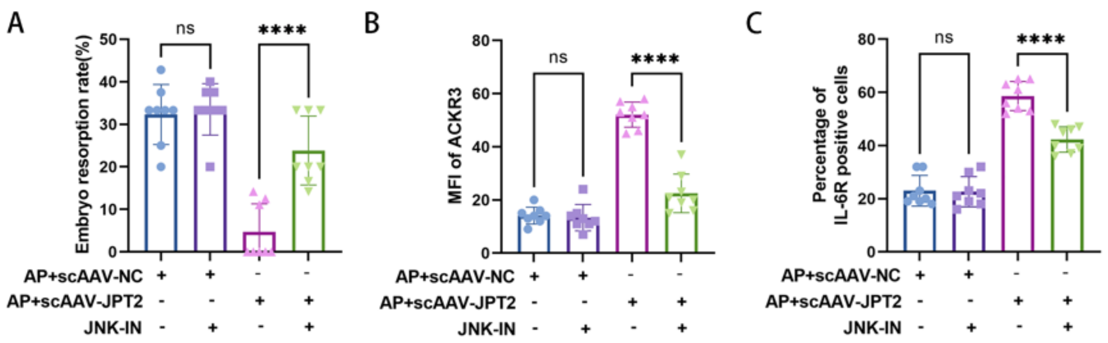

Figure S17. Activation of JNK signaling is the key to the treatment of RSA via JPT2. (A) Quantitative values of embryo resorption rate (each group: n=8). **(B)**Quantification values of ACKR3 MFI at the placental interface in mice (each group: n=8). **(C)** The percentage of IL-6R positive cells in decidual macrophages of mice (each group: n=8). Error bars indicate the SD of the mean. One-way ANOVA was used to compare differences between multiple groups. ****P < 0.0001, ns: not significant.

Supplementary tables

Table S1. Characteristics of women from the HC and RSA groups.

	HC groups（n=30）	RSA groups(n=30)	*P* value
Age (years)	30.13±3.13	29.93±3.02	0.8025
BMI (kg/m^2)	21.46±1.16	21.56±1.08	0.7266
Gestation age (weeks)	7.46±1.22	7.73±1.08	0.3748
Number of miscarriages	0.13±0.34	2.96±0.85	< 0.0001

HC, healthy control; RSA, recurrent spontaneous abortion; BMI, body mass index *P < 0.05

Table S2. The primer sequences used in the study.

Primer	Forward	Reverse
Human-CD68	CGAGCATCATTCTTTCACC AGCT	ATGAGAGGCAGCAAGATGGAC C
Human-CD86	CCATCAGCTTGTCTGTTTCA TTCC	GCTGTAATCCAAGGAATGTGGT C
Human-IL-1β	ATGATGGCTTATTACAGTGG CAA	GTCGGAGATTCGTAGCTGGA
Human-TNF-α	TCTCGAACCCCGAGTGACA A	TGAAGAGGACCTGGGAGTAG
Human-CD206	AGCCAACACCAGCTCCTCA AGA	CAAAACGCTCGCGCATTGTCC A
Human-CD163	CCAGAAGGAACTTGTAGCC ACAG	CAGGCACCAAGCGTTTTGAGC T
Human-IL-10	TCTCCGAGATGCCTTCAGC AGA	TCAGACAAGGCTTGGCAACCC A
Human-TGF-β2	CAGCACACTCGATATGGAC CA	CCTCGGGCTCAGGATAGTCT
Human-IL-6	AGACAGCCACTCACCTCTT CAG	TTCTGCCAGTGCCTCTTTGCTG
Human-CCL5	CCTGCTGCTTTGCCTACATT GC	ACACACTTGGCGGTTCTTTCGG
Human-IL-1α	TGGTAGTAGCAACCAACGG GA	ACTTTGATTGAGGGCGTCATTC
Human-CXCL-12	CTCAACACTCCAAACTGTG CCC	CTCCAGGTACTCCTGAATCCAC
Human-CXCL-1	AGCTTGCCTCAATCCTGCA TCC	TCCTTCAGGAACAGCCACCAG T

Human-IL-37	TCAGCCTCTGCGGAGAAAGGAA	GCGTGCTGATTCCTTTTGGGCA
Human-CXCL-6	GGGAAGCAAGTTTGTCTGGACC	AAACTGCTCCGCTGAAGACTGG
Human-CXCL-8	GAGAGTGATTGAGAGTGGACCAC	CACAACCCTCTGCACCCAGTTT
Human-IL-33	GCCTGTCAACAGCAGTCTACTG	TGTGCTTAGAGAAGCAAGATACTC
Human-Cs	TGCTTCCTCCACGAATTTGAAA	CCACCATACATCATGTCCACAG
Human-β-actin	CATGTACGTTGCTATCCAGGC	CTCCTTAATGTCACGCACGAT

Cs, Citrate synthase

Table S3. The specific antibodies used for western blotting, immunohistochemistry, immunofluorescence staining, co-immunoprecipitation, and flow cytometry.

Antibodies	Identifier	Source
Anti-LSM12	67029-1-Ig	Proteintech
Anti- HN1L	ab247030	Abcam
Anti- HN1L	ab200587	Abcam
Anti-β-actin	66009-1-Ig	Proteintech
anti-E-cadherin	20874-1-AP	Proteintech
anti-Vimentin	10366-1-AP	Proteintech
anti-ACKR3	DF4859	Affinity Biosciences
anti-IL-6R	DF6466	Affinity Biosciences
anti-p-p38	AF4001	Affinity Biosciences
anti- p38	BF8015	Affinity Biosciences
anti-p-ERK	28733-1-AP	Proteintech
anti-ERK	BF8004	Affinity Biosciences
anti-p-JNK	AF3318	Affinity Biosciences
anti-JNK	66210-1-Ig	Proteintech
anti- Citrate synthase	DF13222	Affinity Biosciences
Anti-mouse IgG (H+L) (DyLight™ 800 4X PEG Conjugate)	5257	CST
Anti-rabbit IgG (H+L) (DyLight™ 800 4X PEG Conjugate)	5151	CST
anti-CD68	28058-1-AP	Proteintech

anti-CD86	ab239075	Abcam
anti-CD206	ab64693	Abcam
anti-F4/80	70076	CST
HRP labeled goat anti-rabbit	5220-0336	SeraCare
PE Anti-human CD86 Antibody	374206	BioLegend
FITC Anti-Mouse CD45 Antibody	E-AB-F1136C	Elabscience
PE Anti-Mouse F4/80 Antibody	E-AB-F0995D	Elabscience
APC Anti-Mouse CD86 Antibody	E-AB-F0994E	Elabscience
APC Anti-Mouse CD206 Antibody	E-AB-F1135E	Elabscience

Materials and methods

Clinical Sample Collection

With ethical approval from the ethics committee of the Renmin Hospital of Wuhan University (WDRY2023-K090), 30 patients with unexplained recurrent spontaneous abortion (RSA group) and 30 patients with normal pregnancies (healthy control, HC, terminated for non-medical reasons) who had abortions were selected for the study, and their villous and decidual tissues were obtained. Exclusion criteria: parental or embryonic chromosomal abnormalities; history of infection; genital tract abnormalities; endocrine abnormalities, and other known causes of miscarriage. Baseline information for both groups of patients is presented in Table S1.

Calcium detection

Ca^{2+} concentration: The Calcium Assay Kit (ab102505, Abcam, UK) was used to detect the concentration of Ca^{2+} in samples. All reagents and samples were mixed according

to the manufacturer's instructions, incubated for 10 min at room temperature, and protected from light. The absorbance values of each well at OD 575 nm were measured in a microplate reader.

Fluorometric labeling of Ca^{2+}: After the samples were washed, 5μM of Fluo 3-AM (IF0150, Solarbio, Beijing, China) was added and incubated at 37°C for 20 min, then 1% fetal bovine serum (FBS) (Gibco, USA) was added and set for 40 min at 37°C. Fluorescence microscopy was used to record the degree of fluorescence.

Enzyme-linked immunosorbent assay (ELISA)

The NAADP ELISA Kit (orb441036, biorbyt, Wuhan, China) and IL-6 ELISA kit (CSB-E04638h, Cusabio, Wuhan, China) were used to detect NAADP and IL-6, respectively, according to the manufacturer's instructions. Briefly, the samples and standards were added to ELISA well plate, incubated at 37°C for 2 h, and then removed the waste liquid, added Biotin-antibody and then incubated at 37°C for 1 h, washed 3 times and then added HRP-avidin, incubated at 37°C for 1 h, washed 5 times, added the color development solution and then incubated at 37°C for 20 min protected from light. Finally, the absorbance value at 450 nm was detected immediately after adding the termination solution.

Cell culture and intervention

The trophoblast cell line HTR8/SVneo (HTR8) and JEG-3 cells were cultured in DMEM-F12 medium (Gibco), and the human monocyte cell line THP-1 cells were

cultured in RPMI 1640 medium (Gibco). All media contained 10% FBS and 1% penicillin-streptomycin (C0222, Beyotime Biotechnology, Wuhan, China). Cells were incubated in a 37 °C full humidity cell culture chamber containing 5% CO_2.

JPT2 overexpression and knockdown lentiviral vectors were purchased from Shanghai GeneChem Co., Ltd. (Shanghai, China). After the lentiviral infection of cells, cell screening was performed using puromycin (ST551-10mg, Beyotime Biotechnology) to obtain stably transfected cell lines.

ACKR3 overexpression plasmid, TPC1 knockdown plasmid, TPC2 knockdown plasmid, and Cs knockdown plasmid were purchased from Vigene (Jinan, Shandong). Transfection was performed using Lipofectamine 2000 Transfection Reagent (11668019, Thermo Fisher Scientific Inc., USA) according to the manufacturer's instructions.

THP-1 cells were incubated in 100 ng/ml phorbol 12 myristate 13 acetate (PMA) (P8139, Sigma, USA) for 24 h to differentiate THP-1 cells into M0 macrophages.

The supernatant of HTR8 and JEG-3 trophoblast cells or M0 macrophages is called conditioned medium (CM).

For IL-6 supplementation, the recombinant human IL-6 (50ng/mL, 200-06, Peprotech, USA) was added to the supernatant of trophoblast for treatment of macrophages for 48 h.

For activation of JNK signaling, Anisomycin (10 μM, HY-18982, MCE, Shanghai, China), an agonist of JNK signaling, was used to treat cells for 48 h.

RNA-sequencing (seq)

Total RNA was isolated from sample using the RNAmini kit (Qiagen, Germany). RNA quality was examined by gel electrophoresis and with Qubit (Thermo, Waltham, MA, USA). Strand-specific libraries were constructed using the TruSeq RNA sample preparation kit (Illumina, San Diego, CA, USA), and sequencing was carried out using the Illumina Novaseq 6000 instrument by the commercial service of Genergy Biotechnology Co. Ltd. (Shanghai, China). The data quality was checked by FastQC v0.11.2. The expression of the transcript was calculated by FPKM (Fragments Per Kilobase of exon model per Million mapped reads) using Perl. Differentially expression transcripts (DETs) were determined using the MA-plot-based method with Random Sampling (MARS) model in the DEGseq package between different time points. Then DETs were chosen for function and signaling pathway enrichment analysis using GO and KEGG databases. The significantly enriched pathways were determined when $P < 0.05$ and at least two affiliated genes were included.

Cell adhesion assay

Fibronectin (F8180, Solarbio, Beijing, China) and pre-warmed denatured 1% bovine serum albumin (BSA) were successively added to the culture plate, cells were spread in each well, and after washing with PBS to remove unadhered cells, the cell count was measured using the CCK-8 kit (CA1210, Solarbio) according to the manufacturer's instructions. The cell adhesion capacity was equal to the absorbance value of adhered cells over the absorbance value of total cells.

Wound healing test

Cells were inoculated in 6-well plates, and after the number of cells reached 90%, a gentle scratch was made with a 200 μL pipette. PBS was used to remove floating debris, and the wound was photographed at 0 h. The cells were then cultured in the serum-free medium. The wounds were photographed at 48 h to measure the extent of wound healing.

Cell invasion assay

Transwell chambers (Corning Inc., Corning, NY, USA) were coated with 80 μL matrigel (BD Biosciences, San Jose, CA, USA) (1:9 dilution). 5×10^4 cells were resuspended in 200μL of serum-free medium and seeded in the upper chamber of the transwell chamber. 600 μL of medium containing 20% FBS was added to the lower chamber. 48 h later, the cells and matrigel in the upper chamber of the transwell chamber were wiped off using a cotton swab. The cells that had invaded the lower chamber were fixed with 4% paraformaldehyde for 15 min, stained with crystal violet for 30 min. Photographs were taken with a light microscope and images were recorded.

RNA extraction and reverse transcription-quantitative polymerase chain reaction (RT-qPCR)

Total RNA was extracted using Trizol (15596026, Thermo Fisher Scientific Inc.). After RNA extraction of samples, the NanoDrop™ One (Thermo Fisher Scientific, USA) was

used to check the RNA concentration and purity. 1 μg of total RNA was reverse transcribed into cDNA strictly according to the instructions of the Reverse Transcription Kit (RR036A, Takara, Shiga, Japan). The level of mRNA expression was measured using the TB Green Premix ExTaq (TliRNaseH Plus) kit (RR420A, Takara). The primer sequences are listed in Table S2.

Untargeted metabolomics

The macrophages were subjected to conditioned media intervention and 1×10^7 cells were collected as a primary sample for untargeted metabolomics. 1000 μL of the extract solution (methanol: acetonitrile: water = 2: 2: 1) containing the isotopically labeled internal standard mixture) was added to the samples. The samples were frozen in liquid nitrogen for 1 min, thawed and vortexed for 30 sec, and the freeze-thaw process was repeated three times. The samples were left to stand in an ice water bath for 10 min and then at -40°C for 1 h. centrifuged at 12000 rpm (RCF=13800(×g), R= 8.6cm) for 15 min at 4 °C. The resulting supernatant was transferred to a fresh glass vial for LC/MS analysis. LC-MS/MS analyses were performed using an UHPLC system (Vanquish, Thermo Fisher Scientific) with a UPLC BEH Amide column (2.1 mm × 100 mm, 1.7 μm) coupled to Q Exactive HFX mass spectrometer (Orbitrap MS, Thermo). The mobile phase consisted of 25 mmol/L ammonium acetate and 25 ammonia hydroxide in water (pH = 9.75) (A) and acetonitrile (B). The auto-sampler temperature was 4 °C, and the injection volume was 2 μL. The raw data were converted to the mzXML format using ProteoWizard and processed with an in-house program developed using R and

based on XCMS for peak detection, extraction, alignment, and integration. Then, metabolites were identified and annotated in conjunction with the Human Metabolome Database (HMDB), the North American Mass Library (MONA), the Metabolite Link (METLIN) public database, and the in-house MS2 database (BiotreeDB, Shanghai). Using SIMCA software (V16.0.2, Sartorius Stedim Data Analytics AB, Umea, Sweden), the data were logarithmically (LOG) transformed plus UV formatted, and then subjected to the automatic modeling analysis of OPLS-DA and Student's t-test, to obtain Variable Importance in the Projection (VIP) and *P*-value, and then corrected for *P*-value by multiple hypothesis testing false discovery rate (FDR). Finally, the data were visualized in RStudio software.

Citric Acid (CA) Content Test

According to the manufacturer's instructions, the Citric Acid (CA) Content Assay Kit (BC2155, Solarbio) was used to detect the content of citric acid. Briefly, cells were collected into centrifuge tubes, centrifuged, and the supernatant was discarded; the cells were crushed by ultrasonic waves after the addition of the extraction reagent to the cells. 10,000g centrifugation was performed at 4℃ for 10 min, and the supernatant was collected. The working solution was prepared according to the manufacturer's instructions, mixed with the collected supernatant and left to stand at room temperature for 30 min, and the absorbance was measured at 545 nm using an enzyme meter.

Western blotting

Total proteins were extracted with RIPA lysis buffer (P0013B, Beyotime Biotechnology), and then the protein concentration was measured using a bicinchoninic acid kit (P0012S, Beyotime Biotechnology). The proteins were separated on SDS-PAGE gels, transferred to PVDF membranes (Millipore, Billerica, MA, USA), blocked in 5% skimmed milk at room temperature for 1 h, and incubated with specific antibodies overnight at 4 °C. The next day, the secondary antibody was added and incubated for 1 h. The immunoreactive proteins on the membrane were visualized using the Odyssey infrared imaging system (LI-COR, Lincoln, NE, USA), and then, the gray value of the protein bands was analyzed using the ImageJ software (NIH, Manassas, MD, USA). All information on the antibodies used is provided in Table S3.

Co-immunoprecipitation (Co-IP)

According to the manufacturer's instructions, the Classic Magnetic Protein A/G IP/Co-IP Kit (YJ201, Epizyme Biotech, Shanghai, China) was used for Co-IP. Briefly, after collection, cells were lysed in cold lysis. Cell lysates were centrifuged to obtain a protein-rich supernatant. Specific antibodies were added to the supernatant and incubated overnight at 4 °C on a rotator. Protein A/G-agarose beads were then introduced into the mixture and rotated for an additional 1 h at room temperature. The collected immunoprecipitation complexes were washed three times. Samples were analyzed by Western blotting to determine protein interactions. All information on the antibodies used is provided in Table S3.

Immunohistochemistry

Immunohistochemistry: Tissues fixed with 4% paraformaldehyde were routinely paraffin-embedded, sectioned, dewaxed, and hydrated, and then subjected to antigen repair. 3% BSA was used for blocking for 30 min, and primary antibodies were added and incubated overnight at 4 °C. The next day, secondary antibodies were added and incubated at room temperature for 1 h. Finally, DAB chromogenic agent was added, and hematoxylin was re-stained. All information on the antibodies used is provided in Table S3.

Immunofluorescence

Immunofluorescence of cells: Cells were fixed with 4% paraformaldehyde at room temperature for 15 min, incubated with 0.3% Triton X-100 at room temperature for 8 min, blocked with 5% BSA at room temperature for 30 min, and incubated overnight with the primary antibodies at 4°C. After incubation, the samples were washed with PBS and incubated with 0.3% Triton X-100 at room temperature for 8 min, incubated with the corresponding fluorescent secondary antibody at room temperature for 30 min in the dark, sealed with an anti-fluorescence quenching solution (including DAPI), and recorded under a fluorescence microscope. Antibodies used are listed in Table S3.

Immunofluorescence of tissues: Paraffin sections were deparaffinized to water, antigenically repaired and blocked with 3% BSA for 30 min. Primary antibody was added and incubated overnight at 4°C. After washing with PBST, the corresponding secondary antibodies were added and incubated in the dark at room temperature for

50 min. Tyramine salt-CY3 or Tyramine salt-488 was added and incubated in the dark at room temperature for 20 min. The nuclei were stained using DAPI and then observed using a microscope system. Antibodies used are listed in Table S3.

EdU

Cell proliferation capacity was measured using the EdU kit (ClickTM, EDU 488, C0071S, Beyotime Biotechnology) according to the manufacturer's instructions. Briefly, cells were incubated with pre-warmed EdU working solution for 2 h, and then fixed with 4% paraformaldehyde for 15 min at room temperature. Cells were incubated with permeabilization solution for 10 min at room temperature. The permeabilization solution was removed and the cells were washed. Cells were added with Click reaction solution and incubated for 30 min at room temperature away from light. The nuclei were stained using DAPI and then observed using a microscope system.

ROS

After sample treatment, samples were washed twice with PBS, incubated with 10 μmol/L 2′,7′-Dichlorofluorescin diacetate (DCF) (CA1410, Solarbio) for 20 min, washed again and then recorded under a flow cytometer or fluorescence microscope.

TUNEL

According to the manufacturer's instructions, the TUNEL kit (C1088, Beyotime Biotechnology) was used to detect apoptosis. Briefly, cells were washed with PBS and

then fixed with 4% paraformaldehyde for 30 min. After washing with PBS, PBS containing 0.3% Triton X-100 was added and incubated for 5 min at room temperature. 50 μL of TUNEL assay solution was added to the samples and incubated at 37°C for 60 min away from light. Finally, the samples were sealed with anti-fluorescence quenching sealing solution and observed under fluorescence microscope.

Animals

CBA/J females, DBA/2 males, and BALB/c male mice were used as in vivo study subjects. CBA/J females were mated with BALB/c males to establish a normal pregnancy (NP) and with DBA/2 males to form an abortion-prone pregnancy (AP). Mice were euthanized on day 11.5 of gestation, and embryo resorption rates were calculated. Animal experiments were approved by the Experimental Animal Welfare and Ethics Committee of the Renmin Hospital of Wuhan University (WDRM20220603B).

For the treatment of NAADP, NAADP-AM (membrane-permeable form of NAADP) was from AAT Bioquest (20999, Sunnyvale, CA, USA). Pregnant mice received intraperitoneal injections of NADDP (0.181 mg/kg) on days 8.5 and 10.5 of gestation.

For blocking of NAADP signaling, pregnant mice received intraperitoneal injections of trans-Ned 19 (20 mg/kg, HY-103316, MCE) on days 8.5 and 10.5 of gestation.

According to the manufacturer's manual, pregnant mice were injected with self-complementary adeno-associated virus 9 (scAAV9) targeting JPT2 (GeneChem Co., Ltd.) via intraperitoneal injections on day 7.5 gestation.

For inhibition of JNK signaling, pregnant mice received intraperitoneal injections of a JNK inhibitor (20 mg/kg, JNK-IN-8, HY-13319, MCE) on days 8.5 and 10.5 of gestation.

Detection of macrophage polarization status by flow cytometry

For cells: After sample treatment, a single cell suspension was collected and incubated with Human TruStain FcX™ (BioLegend, San Diego, CA, USA) at room temperature for 10 min; then, cells are resuspended in FCM staining buffer and then incubated with appropriate fluorochrome-conjugated antibodies for staining. A flow cytometer was used to determine the mean fluorescence intensity of the sample. All information about the antibodies used is provided in Table S3.

For tissues: Mice placental tissues were obtained and quickly washed in ice-cold PBS, minced, and digested in RPMI 1640 supplemented with collagenase type IV (BS165, Biosharp, Anhui, China) and DNase I (BS137, Biosharp) for 2 h. The cell suspension was filtered through a 100 μm cell filter and centrifuged to collect the cells, which were then de-erythrocyted by erythrocyte lysis solution (BL503A, Biosharp). Cells are resuspended in FCM staining buffer and then incubated with appropriate fluorochrome-conjugated antibodies for staining. A flow cytometer was used to determine the mean fluorescence intensity of the sample. All information about the antibodies used is provided in Table S3.

Statistical analysis

All in vitro experiments were repeated at least three times. All data are expressed as mean ± standard deviation (SD) of the mean. All statistical analyses were performed using GraphPad Prism 9.00 (GraphPad Inc., La Jolla, CA, USA). Student's t-test was used to assess differences between the two groups, and one-way ANOVA was used to compare differences between multiple groups. Pearson correlation analysis was used to analyze the correlation between the two factors. P value < 0.05 was considered statistically significant. *$P < 0.05$, **$P < 0.01$, ***$P < 0.001$, and ****$P < 0.0001$, ns= not significant.
